# Design of an injectable, self-adhesive, and highly stable hydrogel electrode for sleep recording

**DOI:** 10.1016/j.device.2023.100182

**Published:** 2023-12-05

**Authors:** Ju-Chun Hsieh, Weilong He, Dhivya Venkatraghavan, Victoria B. Koptelova, Zoya J. Ahmad, Ilya Pyatnitskiy, Wenliang Wang, Jinmo Jeong, Kevin Kai Wing Tang, Cody Harmeier, Conrad Li, Manini Rana, Sruti Iyer, Eesha Nayak, Hong Ding, Pradeep Modur, Vincent Mysliwiec, David M. Schnyer, Benjamin Baird, Huiliang Wang

**Affiliations:** 1 Department of Biomedical Engineering, The University of Texas at Austin, Austin, TX 78712, USA; 2 Department of Neurology, The University of Texas at Austin, Austin, TX 78712, USA; 3 Department of Psychiatry, The University of Texas Health Science Center at San Antonio, San Antonio, TX 78229, USA; 4 Department of Psychology, The University of Texas at Austin, Austin, TX 78712, USA; 5 Lead contact

## Abstract

High-quality and continuous electroencephalogram (EEG) monitoring is desirable for sleep research, sleep monitoring, and the evaluation and treatment of sleep disorders. Existing continuous EEG monitoring technologies suffer from fragile connections, long-term stability, and complex preparation for electrodes under real-life conditions. Here, we report an injectable and spontaneously cross-linked hydrogel electrode for long-term EEG applications. Specifically, our electrodes have a long-term low impedance on hairy scalp regions of 17.53 kΩ for more than 8 h of recording, high adhesiveness on the skin of 0.92 N cm^−1^ with repeated attachment capability, and long-term wearability during daily activities and overnight sleep. In addition, our electrodes demonstrate a superior signal-to-noise-ratio of 23.97 decibels (dB) in comparison with commercial wet electrodes of 17.98 dB and share a high agreement of sleep stage classification with commercial wet electrodes during multichannel recording. These results exhibit the potential of our on-site-formed electrodes for high-quality, prolonged EEG monitoring in various scenarios.

## In brief

AIRTrode features preparation-free, tunable mechanical and electrical properties and superior adhesiveness to achieve a variety of biopotential recording applications. AIRTrode demonstrates a higher signal-to-noise ratio (SNR) than commercial EEG gel together with excellent long-term on-skin stability. The capability of AIRTrode to capture brain activity with higher signal quality and a significantly longer time span than commercial EEG gel holds significant potential for personalized healthcare applications. Our AIRTrode technology has the potential to minimize costs and enhance long-term health outcomes through tailored monitoring and interventions.

## Graphical Abstract

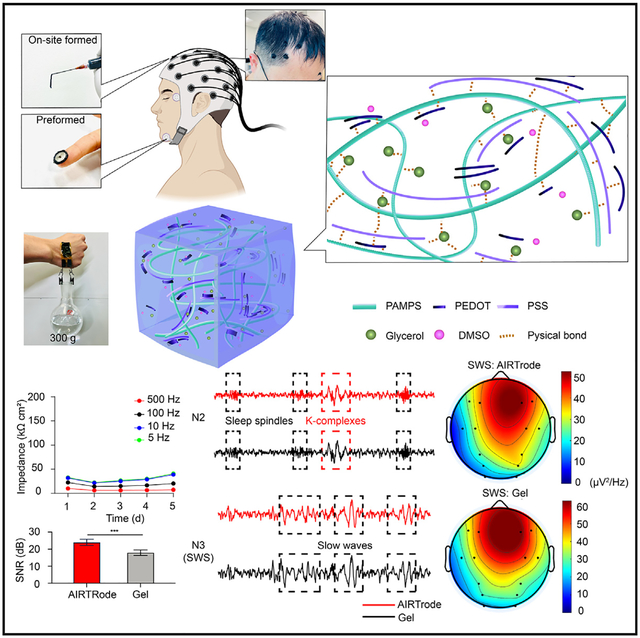

## INTRODUCTION

High-quality and continuous electroencephalogram (EEG) monitoring plays an important role in sleep research and medicine.^1^ EEG recording during sleep enables the identification and monitoring of different stages of sleep and contributes to the diagnosis of sleep disorders.^2–4^ Furthermore, interventions based on sleep EEG can potentially decrease sleep inertia through strategic awakening during shallow sleep stages^5^ and enhance memory through stimulation during the slow-wave stage.^6,7^ These advancements highlight the significant contributions of sleep EEG to improving our understanding of brain function and promoting overall well-being and performance. To enhance access to these applications, a system needs to be easy to operate, robust, and long-term stable. Specifically, this would require the interface to demonstrate (1) a high signal-to-noise ratio (SNR) over extended periods, (2) high adhesion force and conformability to guarantee the connection, and (3) ease of use. The commercial EEG gel that most of the current EEG recording hardware technologies use is easy to use. However, this gel suffers from two key limitations: (1) signal-quality degradation over time as the gel dries out and (2) fragile connections between the conductive gel and the recording technology. These issues compromise the reliability and accuracy of EEG data over time. Therefore, a reliable acquisition platform for EEG is urgently needed. Some high-performance sleep EEG recording electrodes have been reported to offer low impedance and good stability for long-term forehead EEG^8^ and polysomnography (PSG).^9^ However, the reported electrodes cannot be used for the hair-covered region and do not have adhesion to the skin.^8,9^ Hence, a solution for EEG recording from the hair-covered region is still needed. Recently, high-performance conductive hydrogels have been regarded as a promising alternative material for long-term EEG applications^10–18^ because of their ability to penetrate through hair, high adhesion, excellent conformability to the skin, and long-term recording stability.

Conductive soft hydrogels, usually used as pre-formed electrodes, have addressed the issue of commercial EEG gel, such as spreading and drying during EEG recordings.^10,15,18,19^ Moreover, these pre-formed hydrogels are convenient for users because they do not require complex preparation prior to use. However, the pre-formed morphologies of these hydrogels still vastly limit their capability to conform to the geometry of the hairy scalp, resulting in the requirements of specific shapes and external pressing forces to establish reliable contact between the hydrogels and the scalp to reduce the interfacial mismatch and the high contact impedance. On the other hand, on-site-formed electrodes, including injectable and on-skin gelled hydrogels, can penetrate through the hair more effectively, enhancing conformability with the scalp compared with pre-formed electrodes.^11,16,17^

In the field of on-site-formed EEG electrodes, Li et al.^11^ have reported an *in situ* polymerized polyacrylamide/polyvinyl alcohol superporous hydrogel that could release saline from the electrode pillar to achieve low interfacial impedance for EEG-based brain-computer interface (BCI) recording continuously with a robust signal for up to 6 h. Despite the promising results regarding longevity and convenience, the electrodes have no intrinsic adhesiveness, resulting in the need for external support to secure a robust connection. Very recently, Luo et al.^16^ have reported an on-skin instant gelled hydrogel achieved by adding MXene as a cross-linking reagent, resulting in ultralow interfacial impedance. The hydrogels demonstrated good signal quality and moderate adhesion force on the scalp without external fixation for BCI applications.^16^ However, the stability of the SNR over extended periods was not reported. Another on-site gelled hydrogel was reported by Wang et al.;^17^ their hydrogel’s thermally responsive, reversible liquid-gel transition properties allow on-site gelation after thermal treatment before use. The authors demonstrated a robust interface for the system and up to 48 h of operational time for a BCI application. However, the temperature-dependent nature of the phase of the hydrogel made it challenging to precisely control the shape and size of the hydrogel for high-spatial-resolution applications. Furthermore, all of the above EEG measurements were performed while the subjects were awake and in a static position. Hence, it might not accurately reflect real-life scenarios, where significant non-stationarity is often present in recordings. Additionally, all of these reported on-site-formed electrodes require treating the precursor with electrolyte,^11^ a cross-linking reagent,^16^ or heat^17^ before use, which can be inconvenient for users, particularly in home-use systems. So far, it is still challenging to simultaneously achieve a high-quality signal, long-term stability, high on-skin adhesion, and hassle-free pre-use preparation in on-site-formed electrodes.

In this study, we designed an adhesive, injectable, room-temperature (RT) spontaneous cross-linked electrode (AIRTrode) to realize high-quality and long-term EEG recording in the hairy area of the scalp. The reported hydrogel can realize injectable and pre-formed biopotential electrodes to facilitate a variety of use scenarios by simply adjusting its composition ratio. The elastic modulus of the AIRTrode could be easily modulated from 17.1 ± 2.0 to 47.8 ± 11.6 kPa by varying the proportion of its constituent components, enabling its application for injectable electrodes. In addition, the AIRTrode has high adhesiveness to various substrates, including glass (0.98 ± 0.08 N cm^−1^), copper (0.59 ± 0.08 N cm^−1^), and skin (0.92 ± 0.21 N cm^−1^), even after 20 attachment/detachment cycles. The AIRTrode can either replace commercial EEG gels in current EEG recording settings to realize robust and long-term EEG monitoring applications or be used as a stand-alone electrode for integration into pre-assembled wearable electronics because of its tunable mechanical properties. Most importantly, the AIRTrode exhibited superior interfacial electrical impedance characteristics of 17.50 ± 9.0 kΩ and superior stability during continuous on-skin wearing for 5 days. To demonstrate the long-term stability, adhesiveness, and high signal quality of our electrodes in the hairy regions on the scalp, we performed overnight sleep EEG monitoring over 8 h and demonstrated AIRTrode’s excellent stability over 8 h with a higher SNR than the commercial EEG gel electrodes. Finally, we demonstrate that our AIRTrodes and the commercial gel electrodes exhibit significant agreement with the overnight sleep stage classification (r = 0.91 ± 0.06, κ = 0.84 ± 0.06, n = 3). The results of this study fully demonstrate the effectiveness of our AIRTrode during prolonged EEG monitoring periods, enhancing its potential for extended utilization in non-clinical environments, such as home or work settings. The overall comparison between our AIRTrode and other hydrogel-based electrodes for long-term EEG applications is illustrated in Table S1 and Figure S1. From Table S1, it is clear that our AIRTrode stands out as the first reported electrode to successfully simultaneously achieve low on-skin impedance, high on-skin adhesiveness, long-term stability, and a high-quality signal with hassle-free pre-use preparation.

## RESULTS

### Design of the AIRTrode hydrogel

**Figure 1.**
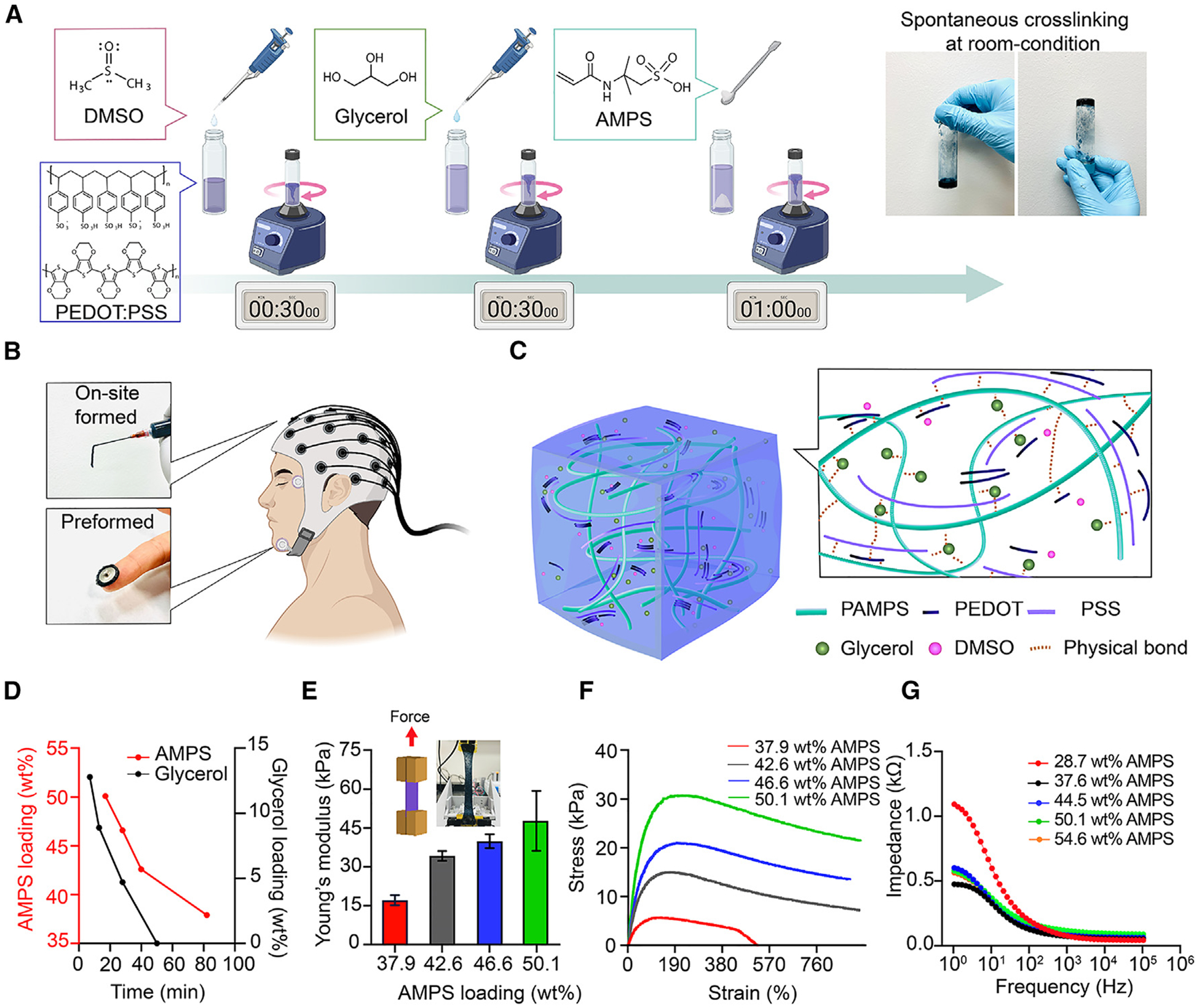
Schematic diagram and materials characterization of the AIRTrode (A) Schematics of the fabrication process of the AIRTrode and the chemical structure of its components. The AIRTrode precursor is prepared by sequentially mixing pristine PEDOT:PSS with DMSO, glycerol, and AMPS, followed by allowing the mixture to sit at room temperature for a specified duration to cross-link without the requirement of external treatment or agents. (B) Pictures of the fabricated AIRTrode with different composition ratios to form an on-site formed (injectable) electrode or pre-formed (self-contained and preshaped) electrode. (C) Schematic of the AIRTrode matrix. The hydrogel network is primarily formed by the physical bonds between glycerol, PAMPS (polymerized AMPS), and PEDOT:PSS. (D) The correlation between the cross-linking time of the AIRTrode and the loading of AMPS and glycerol is examined. Glycerol loading remained consistent at a 10% weight ratio relative to PEDOT:PSS while adjusting AMPS loadings, and AMPS loading stayed consistent at a 100% weight ratio relative to PEDOT:PSS while adjusting glycerol loadings. (E) The correlation between Young’s modulus of the AIRTrode and the AMPS loading. (F) Stress-strain curves of AIRTrode with different AMPS loadings. The effective original length of the samples was 15 mm. (G) EIS test results of AIRTrode with different AMPS loadings. The on-site-formed and the pre-formed electrodes were fabricated based on these two AMPS loadings. This figure was partially created with BioRender.

Our injectable hydrogel comprised 2-acrylamido-2-methylpropane sulfonic acid (AMPS), poly(3,4-ethylenedioxythiophene) polystyrene sulfonate (PEDOT:PSS), dimethyl sulfoxide (DMSO), and glycerol, and we will explain the rationale for each of the components. AMPS has received significant attention in bioelectronics for its high water absorption capability,^20^ high ionic conductivity,^21^ and biocompatibility.^22–24^ These allow the hydrogel to demonstrate a modulus similar to soft biological tissues^25–27^ (1–100 kPa) while possessing high ionic conductivity to reduce the interfacial mismatch as an electrode. To provide both high electrical and ionic conductivity in a variety of biopotential recording applications, PEDOT:PSS was selected based on its exceptional electrical conductivity and tunable mechanical properties.^28^ DMSO and glycerol were incorporated as secondary dopants to modulate the electrical and mechanical properties of PEDOT:PSS. The high dipole moment of DMSO enables it to interact with PEDOT:PSS to alter the original PEDOT:PSS core-shell structure into elongated, linear chains.^29,30^ Glycerol has been reported to improve water retention capability^10,15^ and stretchability^31^ when incorporated into hydrogels. Furthermore, the hydroxyl groups in glycerol can form hydrogen bonds with amide and hydroxyl groups in the stratum corneum to improve the adhesion force between the hydrogel and human skin. This study describes a simple and efficient method to fabricate a hydrogel composed of PEDOT:PSS/AMPS/DMSO/glycerol that is spontaneously cross-linked, stretchable, and self-adhesive for long-term EEG applications. The detailed fabrication process is detailed under experimental procedures, but the summary of the fabrication process steps is described here and illustrated in Figure 1A. First, DMSO is added to PEDOT:PSS, followed by addition of glycerol and then addition of the AMPS monomer. For each addition, a vortex mixer is used to mix the blend evenly. Finally, the resulting mixture is poured into a pre-shaped 3D-printed mold to allow spontaneous gelation at room temperature. Upon completion of the gelation process, the hydrogel can be molded into various shapes and sizes to accommodate a wide range of applications, as shown in Figure 1B. The formation of the AMPS-based hydrogel at room temperature is tentatively attributed to the existence of the sulfonic acid group in AMPS. The highly dissociative sulfonic acid group could readily dissociate in water to release the counterions (H^+^). The released hydrogen ions (H^+^) can promote the polymerization of AMPS to form poly-AMPS (PAMPS) through the catalytic effect.^32^ Moreover, the dynamic hydrogen bonds formed between inter-chain carbonyl, amino, and sulfonic groups could act as physical cross-links, forming a self-cross-linked PAMPS hydrogel.^32,33^ The formation of hydrogen bonding is verified by Fourier transform infrared (FTIR) spectroscopy. As shown in Figure S2, the AIRTrode displayed a distinct infrared (IR) band attributed to intermolecular hydrogen bonds, which peaked in the 3,290 cm^−1^ regions.^34^ The band observed at 1,660 cm^−1^ corresponded to the C=O stretching vibration in AMPS.^35^ However, a noteworthy observation was that the AIRTrode exhibited a shift in the C=O stretching vibration from 1,660 cm^−1^ to 1,620 cm^−1^. This shift was attributed to the formation of C=O⋯HO-type hydrogen bonds.^36^ These findings demonstrate the significant influence of hydrogen bonds on the intermolecular interactions within the hydrogel structure. Incorporating PEDOT:PSS in the AMPS polymerization system can also increase the amount of reactive sulfonic acid groups, particularly when the ionic bonds between PEDOT chains and PSS chains are interrupted by the presence of DMSO and glycerol.^31,37^ As a result, it promotes the spontaneous polymerization of AMPS under milder temperature conditions compared with previous reports.^32^ Furthermore, increasing the number of sulfonic and hydroxyl groups is expected to increase the formation of inter-chain physical bonds and, hence, will lead to a shorter required time for polymer network formation. This is consistent with the experimental observation of the reduced gelatinization time with increased AMPS loadings and glycerol loadings (Figures 1C and 1D).

### Mechanical and electrical properties of the AIRTrode

Tensile stress tests were conducted to investigate the elastic modulus tunability of the AIRTrode. Figure 1E shows a hydrogel sample image during experiments under tensile stress. The elastic modulus, or Young’s modulus, can be adjusted by varying the AMPS ratio within the hydrogel. Figure 1F demonstrates how AMPS loading impacts the elastic modulus of the hydrogel. A range of AMPS loadings was tested, from 37.9 wt % to 50 wt %. The highest elastic modulus value was recorded at 50.1 wt % AMPS loading (47.8 ± 11.6 kPa), while the lowest was recorded at 37.9 wt % AMPS loading (17.1 ± 2.0 kPa). The elastic modulus gradually increased with increasing AMPS loading, with values of 39.9 ± 2.7 kPa for 46.6 wt % AMPS loading and 34.2 ± 1.9 kPa for 42.6 wt % AMPS loading. The tissue-like elastic modulus^25–27^ (1–100 kPa) of the AIRTrode makes it an ideal material for use as an interface in human-machine interfaces. Additionally, the increase in AMPS loading also affects the toughness of the hydrogels. As shown in Figure 1F, the stress-strain curves demonstrate that the elongation at break for samples with 42.6, 46.6, and 50.1 wt % AMPS loading was greater than 700%, while the elongation at break for the samples with 37.9 wt % AMPS loading was around 350%. Overall, all samples demonstrated significantly greater stretchability than the requirement for highly stretchable epidermal electronic devices for mechanically compliant human skin^38^ (ε > 100%, where ε is the strain), making the AIRTrode material well suited for use in stretchable bioelectronics applications while maintaining its conformal contact with the skin.

To determine the optimal AMPS content for the AIRTrode with regard to impedance, hydrogels with different AMPS loadings were investigated. The hydrogels were poured into a 3D-printed tank measuring 20 × 20 × 2 mm^3^, with copper tape connectors used for connection to a potentiostat (SP-300, BioLogic, USA). The hydrogels with AMPS loadings increased from 28.7 to 54.6 wt % and demonstrated impedance reductions from 1,090 to 562 Ω at 1 Hz. (Figure 1G). This is expected with more sulfonic acid groups from PAMPS being ionized and contributing to ionic conductivity. In the low-frequency regimen, all conditions showed lower impedance than the commercial EEG gel (Figure S3A).

The glycerol loading was also investigated because glycerol has been reported to improve the mechanical properties and the conductivity of PEDOT:PSS-based hydrogels. Previous studies have reported that the enhancement in conductivity can be attributed to the screening effect of glycerol, which reduces the Coulombic attraction between positively charged PEDOT and negatively charged PSSH chains.^31,39^ As shown in Figure S3B, increasing the glycerol loading from 0 to 19.6 wt % relative to the total weight of the hydrogel improved the impedance at 1 Hz from 1,350 Ω (0 wt %) to less than 650 Ω for samples with at least 4.7 wt % glycerol loading. No notable difference in the impedance of the hydrogel was observed when the hydrogels had more than 4.7 wt % glycerol loading.

Based on the results of the mechanical and electrical property experiments, the AMPS loading of the optimized AIRTrode was determined to be ~37 wt % and ~45 wt % for use as injectable and pre-formed electrodes, respectively. Glycerol loading of the AIRTrode, in addition to its impact on electrical and cross-linking characteristics, also affects adhesion and stability properties. Therefore, the optimized glycerol loading for the AIRTrode can be determined by specific-use cases.

### Adhesive property of the AIRTrode

**Figure 2.**
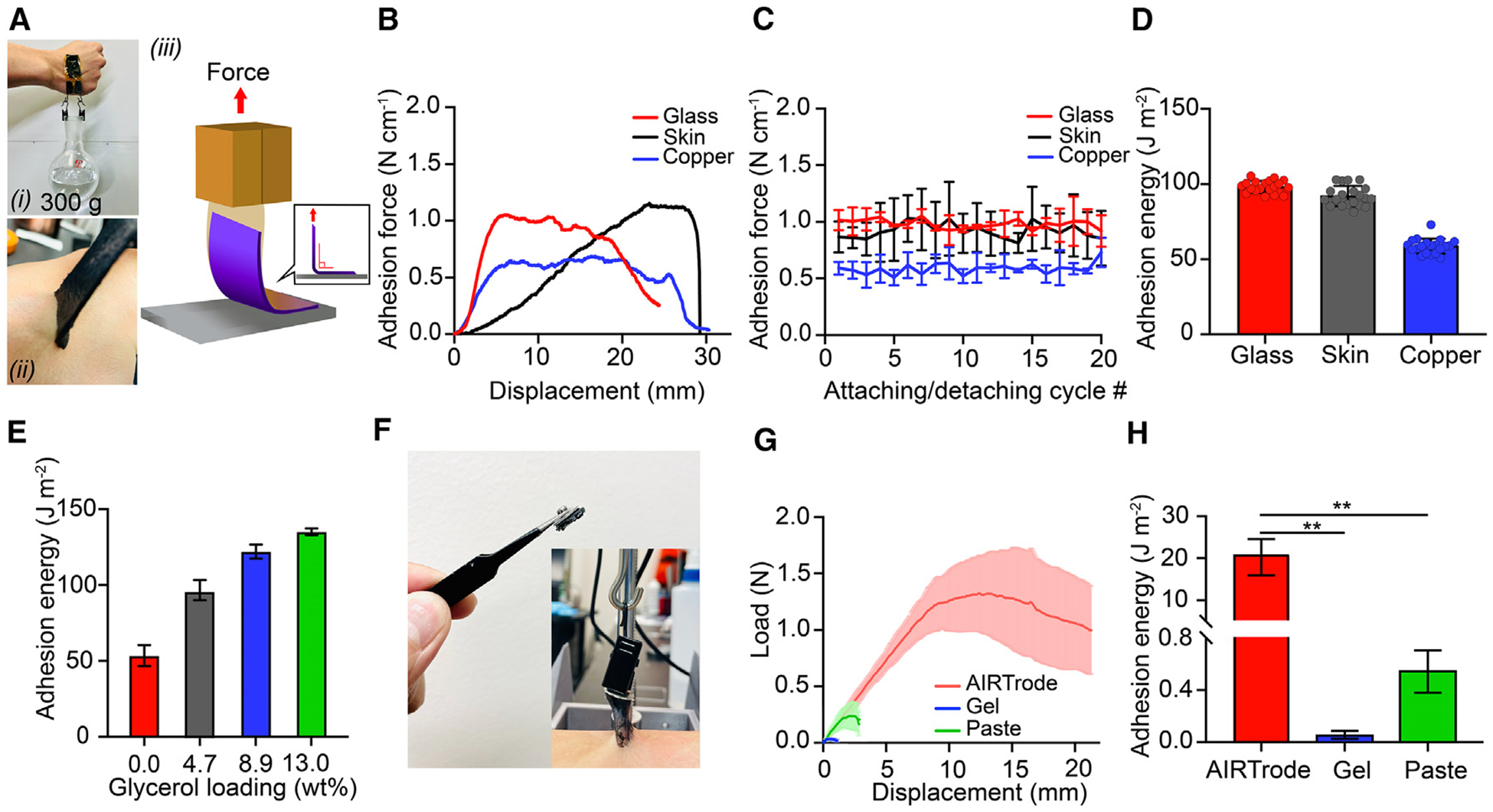
Adhesion strength characteristics of the AIRTrode (A) Image of on-skin adhesion strength demonstration. The AIRTrode sample held a 300 g weight on the back of a volunteer’s hand (i). Also shown is an AIRTrode sample peeled off from a volunteer’s forearm (ii) and the setup of the interfacial adhesion measurements on the skin or glass by the standard 90°-peeling test (iii). (B) Representative adhesion force-displacement curves on glass, skin, and copper. (C) Adhesion force-displacement curves of the 20-cycle attachment/detachment test of AIRTrodes on glass, skin (back of the hand), and copper substrates. (D) The adhesion energy across the 20 cycles of attachment and detachment. (E) The correlation between the glycerol loading and the adhesiveness of the AIRTrodes. (F) Images of the setup of tensile adhesion force tests. A metal plate connector is used for the clip of the mechanical testing system to clasp. The sample to test (AIRTrode, commercial EEG gel, and EEG paste) is sandwiched between the metal plate and the skin. (G) The load-displacement curves obtained from the tensile adhesion tests of the AIRTrode, commercial EEG gel, and EEG paste. (H) The adhesion energy of the AIRTrode, commercial EEG gel, and commercial EEG paste. **p < 0.01, n = 4.

The AIRTrode exhibits exceptional adhesion on various substrates. As depicted in Figure 2Ai, a 20 × 35 × 1.5 mm AIRTrode can remain attached to the skin while carrying a weight load of 300 g. To quantify the adhesion force of the hydrogel, we performed 90°-peeling tests on glass, skin, and copper substrates; this is a widely used technique to assess the adhesion energy of an adhesive patch.^18,40^ The setup of the adhesion tests is illustrated in Figure 2A(iii) and detailed under experimental procedures. We conducted skin adhesion force tests on the back of volunteers’ hands to obtain quantitative data (Figure 2A[ii]). Figure 2B shows the representative load-displacement curves for the hydrogel on different substrates. The adhesion force of the AIRTrode on glass and skin is generally higher than on the copper substrate. The physical bonds formed between the AIRTrode and substrates are responsible for its adhesion force, primarily due to the SO_3_H, -OH, and -CO-NH- groups. These functional groups are capable of forming hydrogen bonds with substrates. For instance, the primary component of glass is silicon dioxide (SiO_2_), which contains numerous available hydroxyl groups (-OH) that can form hydrogen bonds with amide (-CO-NH-) groups in PAMPS, hydroxyl groups (-OH) in glycerol, and sulfonic acid (-SO_3_H) groups in PAMPS and PEDOT:PSS, resulting in strong physical adhesion between the AIRTrode and the glass surface. The same principle applies to the skin. The stratum corneum, the outermost layer of the skin, can also form hydrogen bonds with the AIRTrode because it primarily comprises keratin protein and lipids.^41^ Furthermore, the conformability of the AIRTrode allows the surface of the hydrogel to adapt to the skin’s geography, thereby enhancing the contact area and adhesion between the hydrogel and the skin. To evaluate the repeated attachment and detachment capability of hydrogel in wearable electronics, we conducted the repeated attachment/detachment cycle test. As shown in Figure 2C, the adhesion force values did not decrease even after 20 attachment/detachment cycles. Consistent with the results in Figure 2B, the adhesion force of the hydrogel to glass and skin substrates is comparable. The averaged (n = 4) AIRTrode’s adhesion force to copper is 0.59 ± 0.08 N cm^−1^, while the adhesion force values to glass and skin are 0.98 ± 0.08 and 0.92 ± 0.21 N cm^−1^, respectively. It is worth noting that the standard deviation of the adhesion force on the skin substrate is generally greater compared with the other two substrates. This variation is presumably due to the differences in skin conditions across the volunteer participants. The adhesion energy values across the attachment/detachment cycle are shown in Figure 2D and are calculated using a previously reported equation,^42^ where the adhesive energy (J m^−2^) equals 100 × plateau value of load divided by the width in the 90°-peeling test (N cm^−1^). Figures 2D and S4 show that the adhesion energy values were highly stable on each type of substrate. Particularly in Figure S4, the adhesion energy values for the hydrogel were grouped into four categories based on cycle number, with each group containing five values. The results revealed that the hydrogel exhibited stable adhesion energy values on all substrates, with values ranging from 96.71–100.97 J m^−2^ on glass, 88.18–98.04 J m^−2^ on the skin, and 55.75–60.30 J m^−2^ on copper. Furthermore, it is worth noting that the adhesiveness of the hydrogel can be adjusted by varying the glycerol loading in the composition. As depicted in Figure 2E, the adhesion energy of the AIRTrode on a glass substrate increases with glycerol loading. Specifically, samples without glycerol loading demonstrate the lowest adhesiveness at 53.57 ± 0.07 J m^−2^. In contrast, the adhesiveness of hydrogels increases with 4.7, 8.9, and 13.0 wt % glycerol loading, with corresponding adhesion energy values of 96.58 ± 0.07, 122.02 ± 0.05, and 135.02 ± 0.02 J m^−2^, respectively. This phenomenon can be attributed to the increased number of hydrogen bonds formed between the hydrogel and the substrate, as the number of hydroxyl groups (-OH) in the hydrogel increased with the loading of glycerol.

In addition to the 90°-peeling test, we also performed a tensile adhesion force test to further investigate the interfacial adhesiveness of the AIRTrode as an electrode for wearable electronics. The difference between the 90°-peeling test and the tensile adhesion force test is that the tensile adhesion force test measures the ability of the electrode to maintain its attachment under the stress of tension, such as the pulling force induced to the EEG cap by the users from moving on the pillow during overnight long-term EEG monitoring. This is particularly important in wearable EEG applications, where the electrode needs to withstand the movements and ensure the connection between the wearable device and the skin/scalp. Figure 2F shows an image of a snap clip metal electrode with the AIRTrode and a tensile adhesion test on a volunteer’s arm. The AIRTrode demonstrated superior adhesion force on the skin while also exhibiting good cohesion properties, thereby maintaining interfacial attachment between the skin and wearable device without failure. Upon release from the clip, the electrode quickly reattached to the skin, as shown in Figure S5. This phenomenon is reflected in the large area under the curve (AUC) of the load-displacement curve in Figure 2G, further highlighting the favorable properties of the AIRTrode as an interfacing material in wearable devices. The tensile adhesion force was measured, as shown in Figure S6. The gold-standard commercial EEG gel and commercial EEG paste were also tested to compare the load-displacement curves and the adhesion energy with our AIRTrode, as shown in Figures 2F and 2G, respectively. As shown in Figure 2H, the adhesion energy recorded from the AIRTrode was significantly greater (p < 0.01, n = 4) than the commercial EEG gel and EEG paste used in commercially available EEG systems. The adhesion energy of the AIRTrode, EEG gel, and EEG paste were 20.73 ± 4.40, 0.05 ± 0.03, and 0.54 ± 0.16 J m^−2^, respectively.

### Long-term stability of the AIRTrode

#### Weight loss and electrical impedance stability under open-air conditions

To ensure high signal quality for long-term EEG applications, it is crucial to examine the long-term stability of the AIRTrode. Therefore, we assessed its stability in various conditions, including open-air, on-skin, and on-scalp conditions. Notably, the AIRTrode showed superior stability under all conditions. To assess the long-term stability of the hydrogel under open-air conditions, we monitored its weight loss and impedance for 72 h to provide insights into its water loss and electrical impedance changes. The impedance was measured using a two-electrode method. A fresh pre-formed AIRTrode sample with a diameter of 12 mm was sandwiched between two conductive electrodes (copper tapes on a glass substrate) and connected to a potentiostat to measure the impedance, as shown in Figure S7. The sample was then continuously weighed and measured for weight loss and impedance under open-air conditions (at 22.6°C RT and 45.0% relative humidity [RH]) for 72 h. We evaluated the impact of humidity on electrical impedance by comparing impedance values under various environmental conditions, including ambient conditions (53.6% RH, 22.7°C), high-humidity conditions (83.0% RH, 30°C), and low-humidity conditions (9.5% RH, 23.5°C). Electrochemical impedance spectroscopy (EIS) curves depict the averaged impedance values of a set of electrodes (n = 8) exposed to different humidity levels. As Figure S8 shows, at the same sampling frequency, the differences in measured impedance across humidity conditions are small. Although the impedance difference is higher at 1 Hz, the values are small enough not to affect the electrode-skin interfacial impedance. Furthermore, the measured impedance trend across the frequency band (1–1,000 Hz) is similar under different humidity conditions. These results indicate the operational stability of the AIRTrode under different humidity conditions. The stability of AIRTrode electrodes on sweaty skin was also examined. AIRTrode electrodes were worn on the forearm when the subject went for an hour of outdoor walking at a high environmental temperature (32°C). As depicted in Figure S9, AIRTrode electrodes remained adhered to the skin after sweat was secreted. These results indicate the operational stability of the AIRTrode under different humidity conditions. These collective results support the stability of AIRTrode electrodes in the presence of sweat and in different RH environments.

**Figure 3.**
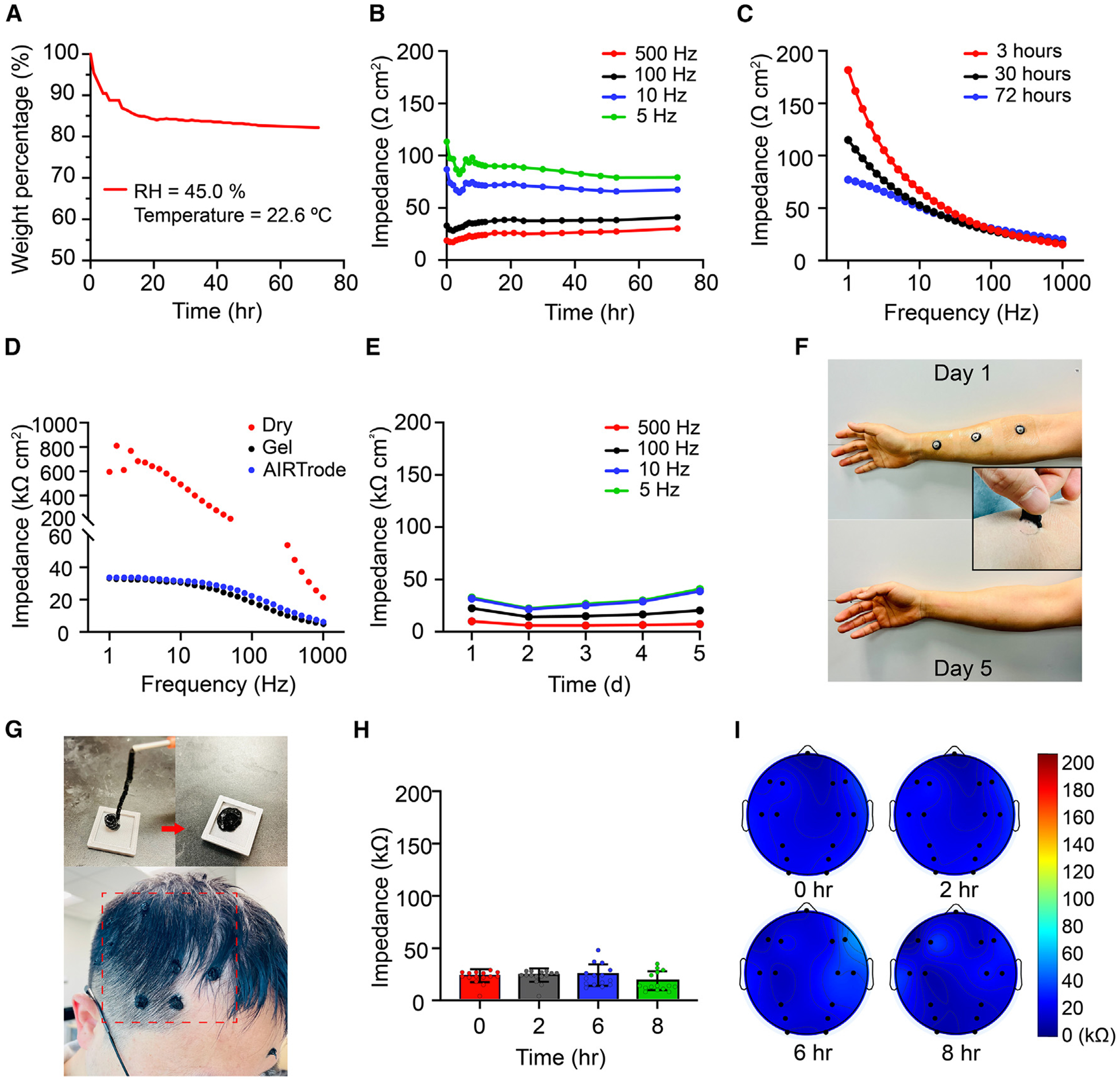
Long-term electrical impedance stability of the AIRTrode (A) The long-term stability of the as-prepared AIRTrode was evaluated by subjecting the sample to open-air conditions at room temperature and moderate humidity. The weight loss of the sample was monitored continuously for over 72 h. (B) The EIS measurements at 500, 100, 10, and 5 Hz across the duration of weight loss monitoring. (C) The EIS sweep was conducted 3, 30, and 72 h after the start of the experiment. (D) EIS of the skin-electrode interface with commercially available dry, commercial EEG gel, and AIRTrode electrodes. The standard three-electrode method was used. (E) The electrical impedance curves of the on-skin skin-electrode interface over 5 consecutive days. Three pre-formed AIRTrodes were applied to the subject’s forearm, and impedance measurements were performed once daily. The impedance values were recorded at 500, 100, 10, and 5 Hz. (F) The placement of the electrodes on the forearm on day 1 (top), the process of removing the electrodes on day 5 (center), and the appearance of the forearm after the removal of the electrodes on day 5 (bottom). (G) The AIRTrode can be extruded onto the scalp using a syringe with a blunt needle, allowing it to form an electrode directly on site. The filament-like extruded hydrogel can seamlessly transform into a complete electrode (AIRTrode) shape upon application to the scalp. (H) The electrical impedance values of the scalp-electrode interfaces were recorded across channels over a specific duration of time. The subjects maintained their regular daytime working schedule throughout the experiment while wearing 16 AIRTrodes on the scalp. (I) The channel-wise view of the scalp-electrode electrical impedance over time.

As depicted in Figure 3A, the majority of the weight loss (9.5 wt %) occurred within the first 5 h after fabrication, which could be attributed to water evaporation from the outer surface of the hydrogel and the formation of a water membrane on the surface of the hydrogel from excess water. The weight loss rate declined in the following 67 h, and the final weight loss under the open-air condition was 17.1 wt %. Similarly, the impedance measurement showed a similar trend, with a decrease in impedance after the fluctuation period, eventually reaching a plateau throughout the remaining time (Figure 3B). The relatively unstable phase of the hydrogel in the first few hours is also evident in the impedance measurements shown in Figure 3B. Additionally, as shown in Figure 3C, there was a more significant difference between the impedance-frequency scan curves at the third and 30th hour compared with the difference between the 30th hour and the final measurement. In addition, Figures S10A–S10C present a direct comparison of the 24-h impedance change between the AIRTrode and the commercial EEG gel under open-air conditions. The results clearly indicate that the AIRTrode exhibits significantly higher long-term electrical stability compared with the commercial EEG gel. The AIRTrode demonstrates a significantly lower impedance change compared with the commercial EEG gel over the course of 24 h across the frequency band from 1–1,000 Hz (paired t test, two-tailed, p < 0.0001), suggesting that the AIRTrode has better long-term stability than the commercial EEG gel.

#### Long-term on-skin impedance stability

On-skin stability was evaluated using a standard three-electrode method,^15^ as illustrated in Figure S11A. The counter electrode (CE), the reference electrode (RE), and the working electrode (WE) were arranged on volunteers’ forearms, with each electrode positioned 7 cm apart from adjacent electrodes. On-skin impedance was monitored continuously for 5 consecutive days, with each participant’s electrode-skin interfacial impedance measured once daily. Participants were permitted to follow regular routines while wearing the electrodes, including exercising. The electrodes were covered with waterproof medical covers during the night to enable showering and prevent detachment during sleep.

The on-skin impedance-frequency scan curves for a commercial EEG dry electrode (labeled “dry” in Figure 3D), a commercial EEG gel electrode (labeled “wet” in Figure 3D), and the AIRTrode electrode (labeled “AIRTrode” in Figure 3D) are compared in Figure 3D. The dry electrode demonstrated significantly higher impedance values across all frequencies than the other electrodes. The AIRTrode electrode showed impedance across frequencies comparable with the commercial EEG gel electrode. Specifically, the impedance of the AIRTrode at 1 Hz and 10 Hz was 33.6 and 31.7 kΩ cm^2^, respectively, while the impedance of the commercial EEG gel at 1 Hz and 10 Hz was 33.0 and 30.4 kΩ cm^2^, respectively. As shown in Figure 3E, the electrode-skin interfacial impedance values of the AIRTrode for the same subject remained stable and below 40.0 kΩ cm^2^ across all frequencies (1–500 Hz) over 5 days Figure S11B presents the averaged impedance measurements obtained from a cohort of participants (n = 6) over 5 days. The impedance values were evaluated at various sampling frequencies; namely, 500 Hz, 100 Hz, 10 Hz, and 5 Hz. Notably, the analysis revealed no statistically significant changes in the average impedance values across different time points. These findings suggest the stability of impedance measurements over time and indicate the consistency of the electrode performance. Notably, no discernible irritation effects were observed among all 6 subjects over the consecutive 5-day period of electrode wearing. Figure 3F provides a visualization of the electrodes on a subject’s arm, showcasing images captured on day 1 (top), on day 5 during removal (inset), and on day 5 after removal (bottom). For a more detailed examination, Figure S12 presents a pair of magnified images depicting the state before and after AIRTrode removal. In these images, the electrode area is highlighted with red dashed circles, revealing that any redness observed in the surrounding area is attributed to the medical adhesive pad rather than the electrode. Notably, after a waiting period of 5 min, no residual redness remains. This collective evidence supports the absence of skin irritation and suggests that the AIRTrode is suitable for applications that require an extended wearing period.

To assess the practicality of utilizing the AIRTrode on the scalp in daily applications, an evaluation of on-scalp impedance stability was conducted over an 8-h period. The results are depicted in Figures 3G–3I. The electrodes were applied to the scalp of the subject through the openings on an EEG cap (actiCAP, Brain Vision) and the active electrode (actiCAP slim electrode, Brain Vision) with a 10-mL Luer-lock syringe with a blunt needle, as illustrated in Figure S13 and Figure 3G, top left. We injected our AIRTrodes on site with high uniformity, as demonstrated in Figure 3G. Initially, the extruded hydrogel appeared filament like (Figure 3G, top left) and then transformed into rounded electrodes after filling the cap (Figure 3G, top right). The bottom image in Figure 3G shows the electrodes’ condition after removing the EEG cap, highlighting their strong attachment to the scalp, even in densely hairy regions. Additional details regarding the electrode placement can be found under experimental procedures. Following electrode application, the subject could resume regular daily activities, except for intense sports or exercises. Impedance values were measured using the built-in impedance measurement function of the EEG amplifier (actiCHamp, Brain Vision) 0, 2, 6, and 8 h post wearing. As shown in Figure 3H, the average impedance values across channels at 0, 2, 6, and 8 h were recorded as 22.33 ± 6.25 kΩ, 22.80 ± 6.34 kΩ, 23.47 ± 10.31 kΩ, and 17.53 ± 9.02 kΩ, respectively. These results demonstrate the excellent stability and resilience of the AIRTrode, which maintained low impedance values throughout the measurement periods despite minor fluctuations due to changes in skin conditions (e.g., sweating) and movements. A more detailed evaluation of impedance value changes across different channels is presented in Figure 3I, showcasing the stability in impedance values for most channels over time. Only a slight impedance increase was observed at channels C4, C6, F4, and F6 at the sixth hour. This may be attributed to reported instances of contact interference by the subject. Contact quality improved by the eighth hour, with impedance values returning to a similar level as the initial measurement.

### Overnight sleep EEG recording application of the AIRTrode

Sleep EEG recording is important for understanding the complexities of sleep patterns, brain activity, and overall sleep quality.^43^ It also provides valuable insights into various sleep stages, such as the slow-wave stage (SWS), rapid eye movement (REM), and wakefulness. The application of sleep EEG includes the investigation of sleep disorders and sleep-related cognitive processes and the evaluation of sleep interventions.^6,44^ To reduce the non-stationarity and noise that will contaminate the data, the capability of a system to maintain the stability of EEG signal quality across long-term EEG monitoring is paramount. Overnight sleep recordings typically span 8 h and involve continuous monitoring of brain activity during different sleep stages and transitions. Ensuring the stability of EEG signal quality throughout the recording duration is crucial for obtaining reliable and accurate data for analysis and interpretation. This section presents the overnight EEG recording capability with AIRTrode. A commercial EEG gel was used to benchmark the performance of the AIRTrode.

#### Overnight EEG signal quality and stability of the AIRTrode

**Figure 4.**
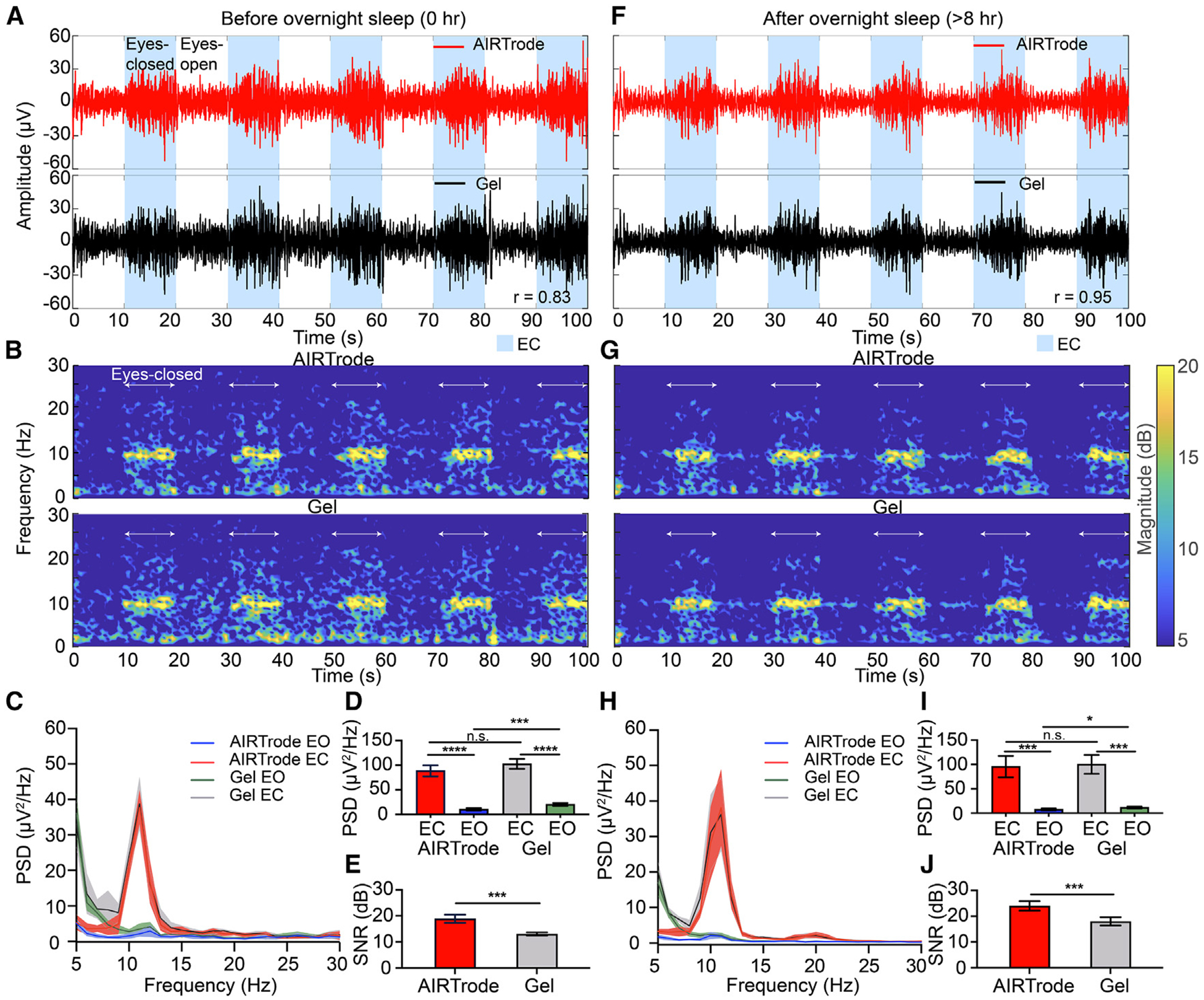
Comparison of Long-term EEG signal quality between AIRTrodes and the commercial EEG gel electrodes (A) The time-series EEG signals were recorded from the volunteer by AIRTrode (top) and commercial EEG gel (bottom) during the 100 s of the eyes-open (EO) and eyes-closed (EC) paradigm before overnight sleep. The [1, 30] Hz band-pass filter was applied to remove any direct current (DC) offset. (B) The spectrograms for EEG signals in (A). (C) The PSD analysis of the grand average of all EC and EO epochs for both types of electrodes. The AIRTrode shows an overall lower background signal (frequencies outside of the alpha band [8, 13] Hz) compared with the commercial EEG gel at the specific channel (PO4) before overnight sleep. (D) The mean power spectral density (PSD) analysis derived from the alpha rhythm for the AIRTrode and commercial EEG gel to detect differences in EEG activity between EO and EC periods (****p < 0.0001, n = 5). (E) Signal-to-noise ratio (SNR) comparison between AIRTrodes and commercial EEG gel electrodes. The signal of interest is defined as alpha rhythm, and the background signal is defined as 5–30 Hz, excluding the alpha rhythm. (F) The time-series EEG signals recorded by the AIRTrode (top) and commercial EEG gel (bottom) during the 100 s of the EO-EC paradigm after overnight sleep. The same setting as in (A) was applied. (G) The spectrograms for the EEG signals in (F). Compared with before sleep, the signal quality improved with both types of electrodes. (H) PSD analysis of the grand average of all EC and EO epochs for both types of electrodes. The PSD variation between epochs is greater than before sleep. (I) PSD from the alpha rhythm to show the capability of both types of electrodes to detect differences in EEG activity between EO and EC periods (***p < 0.001, n = 5). (J) SNR comparison between AIRTrodes and commercial EEG gel electrodes after sleep. (***p < 0.001, n = 5).

Due to the superior adhesiveness and deformability, the AIRTrode has excellent conformability with the hairy scalp. This is expected to lead to high and consistent EEG signal quality overnight. To validate this, an eyes-open and eyes-closed task was conducted before (Figures 4A–4E) and after sleep recording (Figures 4F–4J), comparing the EEG signal qualities obtained from the AIRTrode with those from the commercial EEG gel (Figure 4; refer to experimental procedures for sleep EEG recording details). During the eyes-closed periods, the alpha rhythm is expected to increase, reflecting the relaxation state of the brain.^45^ The time-series EEG signal filtered to the range of [1, 30] Hz from both the AIRTrode and the commercial EEG gel prior to sleep recording is shown in Figure 4A. The blue-shaded areas represent the eyes-closed periods. The recorded EEG signals from the AIRTrode and the commercial EEG gel exhibit similar shapes and amplitudes, as indicated by Pearson’s correlation coefficient (r = 0.83). As anticipated, both electrodes demonstrate increased alpha rhythm during the eyes-closed periods, reflecting enhanced synchronization of brain activity associated with relaxation. Additionally, Figure S14A illustrates the time-series alpha band ([8, 13] Hz) power from both AIRTrode and commercial gel electrodes, demonstrating a high correlation coefficient (r = 0.98) between the two types of electrodes. The time-frequency spectrograms (Figure 4B) from both electrodes reveal distinct frequency spectral components between the eyes-open and eyes-closed states.

The grand average power spectral density (PSD) (Figure 4C) of eyes-open and eyes-closed states from both electrodes, calculated based on 5 epochs (refer to the experimental procedures), shows similar amplitudes in the alpha band components during eyes-closed states. However, the commercial EEG gel captures more background signal than the AIRTrode during eyes-open states, attributed to higher noise being more prominent at the channels in the commercial EEG gel due to the non-stationarity of the contact area induced by gravity, fluidity, and the lack of adhesiveness. The alpha band PSD of both electrodes and states is illustrated in Figure 4D. Significant differences are observed between eyes-open and eyes-closed states for both electrodes (two-tailed t test, p < 0.0001, n = 5). Comparing the PSD of the electrodes, there is no significant difference in the alpha band PSD during eyes-closed states between the AIRTrode and the commercial EEG gel (two-tailed t test, p = 0.07, n = 5). However, a statistically significant difference is observed in the alpha band PSD during eyes-open states between the AIRTrode and the commercial EEG gel (two-tailed t-test, p < 0.001, n = 5). Furthermore, Figure 4E presents both electrodes’ mean SNR during eyes-closed states (for details regarding SNR calculations, see experimental procedures) The AIRTrode demonstrates a significantly higher SNR (18.91 ± 1.57 decibels [dB]) compared with the commercial EEG gel (13.05 ± 0.6 dB) due to its capability to record signals with lower background noise (two-tailed t test, p < 0.001, n = 5).

The same analyses were performed on the eyes-open-closed task after the sleep recording (Figures 4F–4J). The filtered time-series EEG signal and the alpha band power are shown in Figures 4F and S14B, respectively. Again, the recorded EEG signals from both electrodes had excellent agreement in terms of Pearson’s correlation coefficients between the AIRTrode and the commercial EEG gel in the filtered EEG signal (r = 0.95) and the alpha band power (r = 0.99). The SNR values for the AIRTrode (23.97 ± 1.79 dB) and commercial EEG gel (17.98 ± 1.61 dB) electrodes (Figure 4J) increased in comparison with the values prior to sleep (Figure 4E), indicating that the contact qualities are improved for the specific channel after 8 h of sleep for both electrodes. Similar to the situation before sleep (Figure 4E), the AIRTrode again demonstrated a significantly higher SNR compared with the commercial EEG gel electrodes as a result of lower background noise (p < 0.001, n = 5).

#### Overnight sleep staging with the AIRTrode

**Figure 5.**
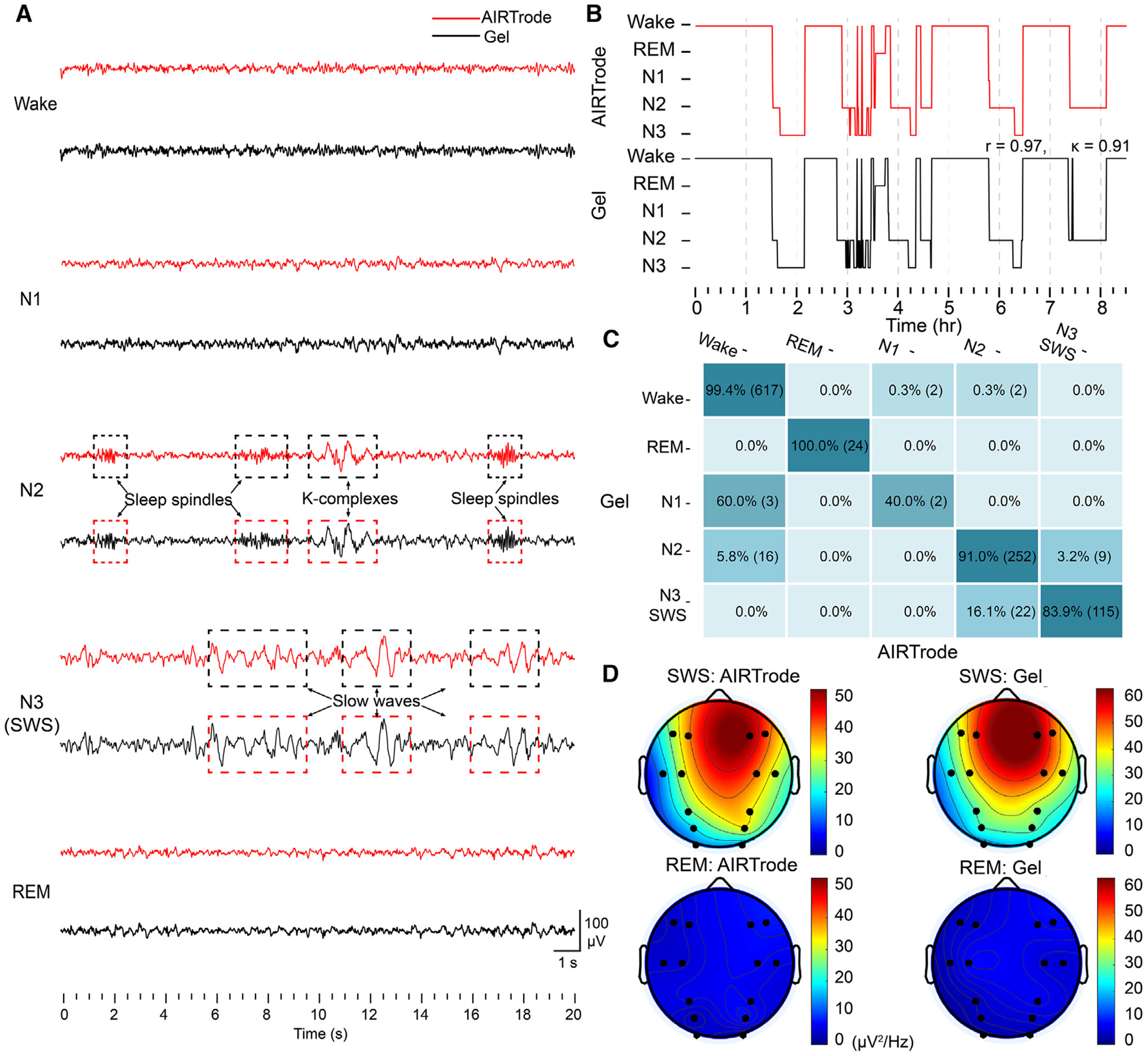
Overnight sleep recording and staging classification with AIRTrode and commercial EEG gel electrodes (A) Representative 20-s samples of raw signals were recorded by the AIRTrode and the commercial EEG gel on the same recording during each sleep stage (N1, N2, N3 [SWS], REM, and wake). The derivations are F3-TP9 for both types of electrodes. The signals are presented between −100 and 100 μV. The sleep spindles and K-complexes are marked with dashed boxes on N2, and the slow waves are marked on N3 (SWS). (B) A set of representative hypnograms from one of the participants, showing the sleep stage classifications with the data recorded from both the AIRTrode and the commercial EEG gel. The two sets of staging results show a significant agreement with each other. Shown are Pearson’s correlation coefficient (r = 0.97) and Cohen’s *κ* (0.91) for the participant. The average Pearson’s correlation coefficient and Cohen’s *κ* across all participants are r = 0.91 ± 0.06 and κ = 0.84 ± 0.06 (n = 3). (C) Confusion matrix for the staging results of sleep data recorded with the AIRTrode versus those recorded with commercial EEG gel. Values are normalized by row with the number of epochs in parentheses. (D) Topographic plots of slow-wave activity (mean PSD in [1, 4] Hz) during SWS (top two plots) and REM stage (bottom two plots) recorded by AIRTrode and the commercial EEG gel.

Figure 5A shows representative samples of EEG signals recorded by the AIRTrode and commercial EEG gel electrodes, obtained from the proximity channel location for overnight sleep (Figure S15), during various sleep stages, including wake, REM, N1, N2, and N3 (SWS). All signal samples were obtained from the F3 channel in the 10–20 commercial EEG system. As shown in Figure 5A, the signals recorded by both types of electrodes exhibit a high degree of similarity. Notably, during the N2 stage, characteristic sleep spindles and K-complexes, two prominent features of this sleep stage, are captured simultaneously by both electrode types. Moreover, the slow waves are shown at the same time points during N3 (SWS) in both datasets. These indicate the effectiveness of AIRTrode in capturing sleep biomarkers.

In Figure 5B, a representative hypnogram of the more than 8 h of recorded overnight sleep EEG data from one of the three subjects is shown, with further details regarding the analysis and system setup under experimental procedures section. The Pearson’s correlation coefficients for sleep staging results obtained from the commercial EEG gel and AIRTrode demonstrate significant agreement across all subjects (r = 0.91 ± 0.06, n = 3). Additionally, Cohen’s *κ* values, another statistical measure used to assess the agreement between two or more systems, indicate almost perfect agreement (κ > 0.8) between the two electrodes across the three subjects (κ = 0.84 ± 0.06, n = 3). The confusion matrix presented in Figure 5C demonstrates excellent agreement in stage classification between the electrodes for the same subject. The most disagreement is at the N1 stage, which can be attributed to the pattern similarity between the N1 stage and the wake stage in EEG signals. It is worth noting that all three subjects experienced a minimal N1 sleep stage during the overnight recording, which aligns with the naturally brief duration of the N1 stage (typically less than 10 min). Thus, this lower percentage of agreement may also, in part, be attributed to the phenomenon of under-sampling, a trend that aligns with findings from previous studies.^46^ The agreement percentages between the two electrodes are 99.4% (617 epochs) for wake, 100% (24 epochs) for REM, 40% (2 epochs; the N1 stage consists of 5 epochs in total) for N1, 91% (252 epochs) for N2, and 83.9% (115 epochs) for SWS. Similar results can be observed in the confusion matrix and hypnogram of the other two subjects, as shown in Figure S16.

Figure 5D exhibits topographic plots of slow-wave activity (mean PSD in [1, 4] Hz) during SWS (top two plots) and REM stage (bottom two plots) recorded by both types of electrodes. As expected, the slow-wave activity is more prominent during SWS compared with REM. Again, the AIRTrodes display recording patterns highly similar to those of the commercial EEG gel electrodes. This result further supports the use of AIRTrodes as a feasible alternative to commercial EEG gel electrodes in long-term EEG applications, such as sleep monitoring, without compromising the excellent signal quality that commercial gel electrodes offer.

## DISCUSSION

We introduce a novel hydrogel (AIRTrode) with spontaneous-cross-linked, injectable, and self-adhesive hydrogel characteristics for long-term EEG recording application. The cross-linking reaction of the AIRTrode could spontaneously occur at room temperature without the presence of any external stimulus or cross-linking reagents. The AIRTrode can be injectable or pre-formed to allow various usage scenarios by simply adjusting its composition ratio. In addition, the AIRTrode possesses tunable electrical and mechanical properties, tunable adhesion toward a variety of substrates, and excellent biocompatibility. The AIRTrode exhibits excellent and stable impedance over time, making it suitable for long-term EEG monitoring. The study also demonstrates the application of the AIRTrode in overnight, multichannel sleep EEG monitoring, with a higher SNR than commercial EEG gel electrodes and significant sleep stage classification agreement with the commercial EEG gel electrodes. We expect our AIRTrode to ultimately be used for realizing home-use, long-term wearable EEG monitoring, and treatment applications. The potential applications can be extended from the closed-loop monitoring and stimulation systems for sleep medicine to broadband neuroscience research and neurological disorders, such as assessing the attention of users^47^ and detecting and characterizing seizure activity, providing spatial and temporal information on brain function during seizure episodes for subsequent intervention decisions.^48,49^

## EXPERIMENTAL PROCEDURES

### Resource availability

#### Lead contact

Further information and requests for resources and reagents should be directed to and will be fulfilled by the lead contact, Huiliang Wang (evanwang@utexas.com).

#### Materials availability

This study did not generate new unique reagents.

#### Data and code availability

The sleep recording and material characterization data have been deposited at Zenodo (https://doi.org/10.5281/zenodo.10059079) and are publicly available as of the date of publication. Any other data reported in this paper will be shared by the lead contact upon request.

All original code has been deposited at Zenodo (https://doi.org/10.5281/zenodo.10059079) and is publicly available as of the date of publication. Any additional information required to reanalyze the data reported in this paper is available from the lead contact upon request.

### Materials

PEDOT:PSS aqueous solution (Clevios PH 1000) was purchased from Heraeus. The concentration of PEDOT:PSS was 1.3 wt % in the solution, and the weight ratio of PSS to PEDOT was about 2.5:1. DMSO and AMPS were purchased from Sigma-Aldrich. Glycerol (>99%) was purchased from Thermo Fisher Scientific. All chemicals were used as received without further purification.

### Preparation of the AIRTrode

First, DMSO is blended with PEDOT:PSS at a 4.5% weight ratio relative to PEDOT:PSS. A vortex mixer (Corning LSE Vortex Mixer, Thermo Fisher Scientific) is used for 30 s to prepare a homogeneous mixture. This mixture is then further blended with at least 4.5 wt % glycerol relative to the total weight (depending on the desired electrode properties) for 30 s using a vortex mixer. The AMPS monomer with 37 wt % (injectable) or 45 wt % (pre-formed) relative to total weight is added to the mixture in the same vortex mixer and mixed for 1 min to obtain a uniform dispersion. The final mixture is then poured into a mold, and the hydrogel is spontaneously formed at room temperature without any external stimulus or cross-linking agent.

### Characterization of mechanical properties

The tensile measurements were performed using a force gauge (FB20N, Torbal) with a custom-designed motorized test stand fabricated through 3D printing (i3 MK3S+, Prusa Research). The force gauge had a maximum load capacity of 20 N, and the uniaxial strain was applied at a controlled ramp rate of 68 mm/min. Prior to the testing, the load cell was calibrated to ensure accurate force measurements. The entire system was controlled using an Arduino board (UNO REV3, Arduino). The samples were prepared to have dimensions of 35 × 20 × 1.5 mm to ensure consistency in specimen size for the experimental evaluation. The effective length of all samples was maintained at 15 mm, with 10 mm being securely attached to non-stretchable tape on the longitudinal ends to facilitate mounting onto the force gauge and the platform.

### Characterization of electrode impedance

To assess the electrical impedance of the AIRTrodes, two methods were employed. First, to measure the impedance of the AIRTrode with varying AMPS and glycerol content and the 24-h period impedance change of the AIRTrode and the commercial EEG gel, a potentiostat (SP-300, BioLogic) was used to measure the impedance values in the range of 1–100k Hz. Each sample was prepared by pouring the precursor into a 3D-printed cuboid tank measuring 20 × 20 × 1.5 mm (Figure S10C) to regulate the shape and volume of each sample. To facilitate connection with the potentiostat, two strips of copper tape measuring 10 × 20 mm were affixed to the opposing side of the tank as extended probes, which were connected to a potentiostat (SP-300) via alligator clips. The measurement setup was also utilized in the 24-h impedance change experiment, as shown in Figure S10. Second, cylindrical AIRTrodes with a diameter of 12 mm and a thickness of 2 mm were sandwiched between two glass plates coated with copper (Figure S7) to investigate the electrical impedance of the AIRTrode interface under prolonged open-air conditions while concurrently monitoring weight loss. Impedance measurements were carried out using a potentiostat (SP-300) within the frequency range of 1–100 kHz. The sample was placed on an analytical balance (PR 124, Ohaus) to quantify changes in weight over time. The copper plates were connected to the potentiostat to facilitate impedance measurements at each designated time point. All measurements conducted in this section were performed on pre-formed AIRTrodes unless otherwise specified.

### Characterization of adhesion properties

The adhesion strength of the AIRTrodes on various substrates was evaluated using 90°-peeling tests and tensile adhesion tests conducted with a dedicated tensile testing system (see Characterization of mechanical properties).

For the cyclic 90°-peeling adhesion test, each AIRTrode sample was prepared with dimensions of 20 mm (width) × 35 mm (length) × 1.5 mm (thickness). Prior to testing, the sample was affixed to a Kapton film backing layer. The hydrogel sample was then gently pressed onto different substrates, including glass, copper, and the back of a volunteer’s hand, for 2 s. A total of 20 attachment/detachment cycles were performed for each sample. All cyclic 90°-peeling adhesion measurements were conducted on pre-formed AIRTrodes. The adhesion energy was determined using the following equation:^42^ adhesive energy (J m^−2^) = plateau value of load/width (N m^−1^).

For the tensile adhesion test, the AIRTrode sample was confined within a 3D-printed ring with a diameter of 12 mm and a thickness of 2 mm during application to the subject’s skin. After the hydrogel was applied, the ring was removed, and a metal connector was attached to the hydrogel by gently pressing it for 2 s. The adhesion energy was calculated based on the area under the load-displacement curve. The control groups, including the commercial EEG gel and paste, underwent the same preparation procedure and testing conditions.

### Stability under various environmental conditions

To evaluate stability in different RH conditions, the electrodes were placed under ambient conditions (53.6% RH, 22.7°C) for 1 h before measuring electrical impedance. After impedance measurements, the electrodes were transferred to an airtight chamber and sat for another 2 h before another impedance measurement. The air in the airtight chamber was controlled to low-humidity conditions. (9.5% RH, 23.5°C) Finally, the electrodes were transferred to high-humidity conditions (83.0% RH, 30°C) and sat for another 2 h before the impedance measurements.

To evaluate stability on sweaty skin, the AIRTrode electrodes were applied to the forearm of the subject, who went for an hour of outdoor walking at a high environmental temperature (32°C).

### Long-term skin-electrode impedance evaluation

The standard three-electrode method^15^ was employed to assess the long-term skin-electrode interfacial impedance. Three pre-formed AIRTrode electrodes were placed on a subject’s forearm and were 7 cm away from the next electrode (Figures 3F and S11) Each electrode had a diameter of 12 mm and a thickness of 2 mm. Each subject wore a set of electrodes for 5 consecutive days without removing them. The electrode-skin interfacial impedance values were measured once daily for each participant. Participants were permitted to follow regular routines with no restrictions, including exercising. The electrodes were covered with waterproof medical covers during the night to enable showering and to prevent detachment during sleep.

### Long-term scalp-electrode impedance evaluation

In this experiment, an EEG signal amplifier with up to 128 channels (actiCHamp plus, Brain Vision) was utilized, along with an EEG cap (actiCAP, Brain Vision) and a set of active electrodes (actiCAP slim electrode, Brain Vision). The AIRTrodes were applied to the subject’s scalp through the cap’s designated openings and the active electrode using a 10 mL Luer-lock syringe fitted with a blunt needle. Sixteen electrodes were employed, including the reference and ground electrodes, with the WE positions selected based on the standard 10–20 EEG system. The WEs comprised F3, F5, C3, C5, P3, PO3, O1, O2, PO4, P4, C6, C4, F6, F4, TP9 (reference), and FPz (ground). This electrode montage effectively covered the frontal, central, and post-occipital areas of the brain, facilitating the evaluation of stability across different areas of the head. Impedance values were measured at four time points: 0 h, 2 h, 6 h, and 8 h. All impedance values were acquired with the built-in impedance measuring function of the EEG amplifier.

### Overnight sleep EEG measurement

#### Sleep EEG setup

3 subjects completed an overnight sleep study at a sleep center while wearing an EEG cap with the setting mentioned under Long-term scalp-electrode impedance evaluation. The two types of electrodes (AIRTrode and the commercial EEG gel) were applied to the proximity locations of each selected channel of the 10–20 EEG system. The resulting montage is shown in Figure S15A. The signals from the two types of electrodes were recorded simultaneously and included in the analysis. Two pairs of pre-formed AIRTrodes were placed on the facial area to record eye movements and muscle movements with horizontal electrooculography (HEOG) and electromyography (EMG) from the temple area and the chin, respectively (Figure S15B).

#### Eyes-open and -closed task EEG signal analysis

The fundamental time-frequency analysis and PSD analysis were performed in MATLAB with toolboxes such as the Signal Processing Toolbox and EEGLAB. For the eyes-open/closed paradigm, the SNRs were computed for the eyes-closed conditions. The electrode located at PO4 was selected for the analysis due to the change in alpha rhythm amplitude being the most prominent around the occipital cortex and surrounding regions.^50,51^ The frequency range of interest was 8–12 Hz (i.e., the alpha band), and the background EEG activities were defined as the EEG spectral responses of 5–30 Hz, while the frequencies of interest were excluded. The SNRs were computed with the following formula: SNR = 10 × log10 (mean [PSD of the frequency range of interest] / mean [PSD of background activities]).

#### Overnight sleep EEG analysis

We assessed (1) the similarity of measured EEG signals between the two types of electrodes, (2) the statistical agreement of the sleep stage hypnograms between the two electrodes within the same subject, (3) the similarity of the slow-wave spatial patterns during different stages between the two types of electrodes. The sleep staging was performed according to American Academy of Sleep Medicine guidelines versus one sleep expert’s manual scoring. Cohen’s *κ* value and Pearson’s correlation coefficient were used to assess the agreement between the hypnograms from the electrodes. Cohen’s *κ* is a statistical measure used to assess the agreement between two or more systems when categorizing or classifying data into mutually exclusive categories. The formula is κ = (P_o_ – P_e_) / (1 – P_e_), where P_o_ is the percentage of observed agreement, and Pe is the expected agreement due to chance. The resulting values should be between −1 and 1. The values can be interpreted as follows: values of 0 or less indicate no agreement and 0.01–0.20 none to slight, 0.21–0.40 fair, 0.41–0.60 moderate, 0.61–0.80 substantial, and 0.81–1.00 almost perfect agreement. All EEG signal data processing, analyses, and visualizations were performed in MATLAB.

## Supplementary Material

1

## Highlights

- Developed a self-adhesive, injectable hydrogel for long-term, high-quality EEG recording
- AIRTrode can continuously record EEG data for over 5 days in a wearable format
- AIRTrode allows hassle-free pre-use preparation (no need for reagents or special treatments)
- AIRTrode outperforms commercial gels in signal-to-noise ratio for EEG recordings

## THE BIGGER PICTURE

Long-term, continuous, and stable electroencephalogram (EEG) monitoring is of paramount importance for neuroscience research, sleep medicine, and patient care. Existing continuous EEG recording technologies suffer from fragile connections and long-term stability issues. Recent developments in this field have made significant progress but still fall short of achieving good on-skin adhesion, long-term stability, high-quality recording, and hassle-free pre-use preparation all at once. In this work, we address these gaps with a hydrogel composition, aiming to fulfill these goals in EEG recordings and pave the way for customizable, wearable, home-use EEG monitoring systems. Further integration of this work with either existing or future long-term EEG monitoring platforms will enable early diagnostics, long-term monitoring, and treatment of brain disorders.
