# Supplementary material for: Design of an injectable, self-adhesive, and highly stable hydrogel electrode for sleep recording
Source: Device. Author manuscript; Available in PMC 2024 Sep 5. (PMC11376683; doi:10.1016/j.device.2023.100182)
Supplement: 1 [file NIHMS1970430-supplement-1.pdf]

DEVICE, Volume *2*

Supplemental information

Design of an injectable, self-adhesive, and highly stable hydrogel electrode for sleep recording

Ju-Chun Hsieh, Weilong He, Dhivya Venkatraghavan, Victoria B. Koptelova, Zoya J. Ahmad, Ilya Pyatnitskiy, Wenliang Wang, Jinmo Jeong, Kevin Kai Wing Tang, Cody Harmeier, Conrad Li, Manini Rana, Sruti Iyer, Eesha Nayak, Hong Ding, Pradeep Modur, Vincent Mysliwiec, David M. Schnyer, Benjamin Baird, and Huiliang Wang

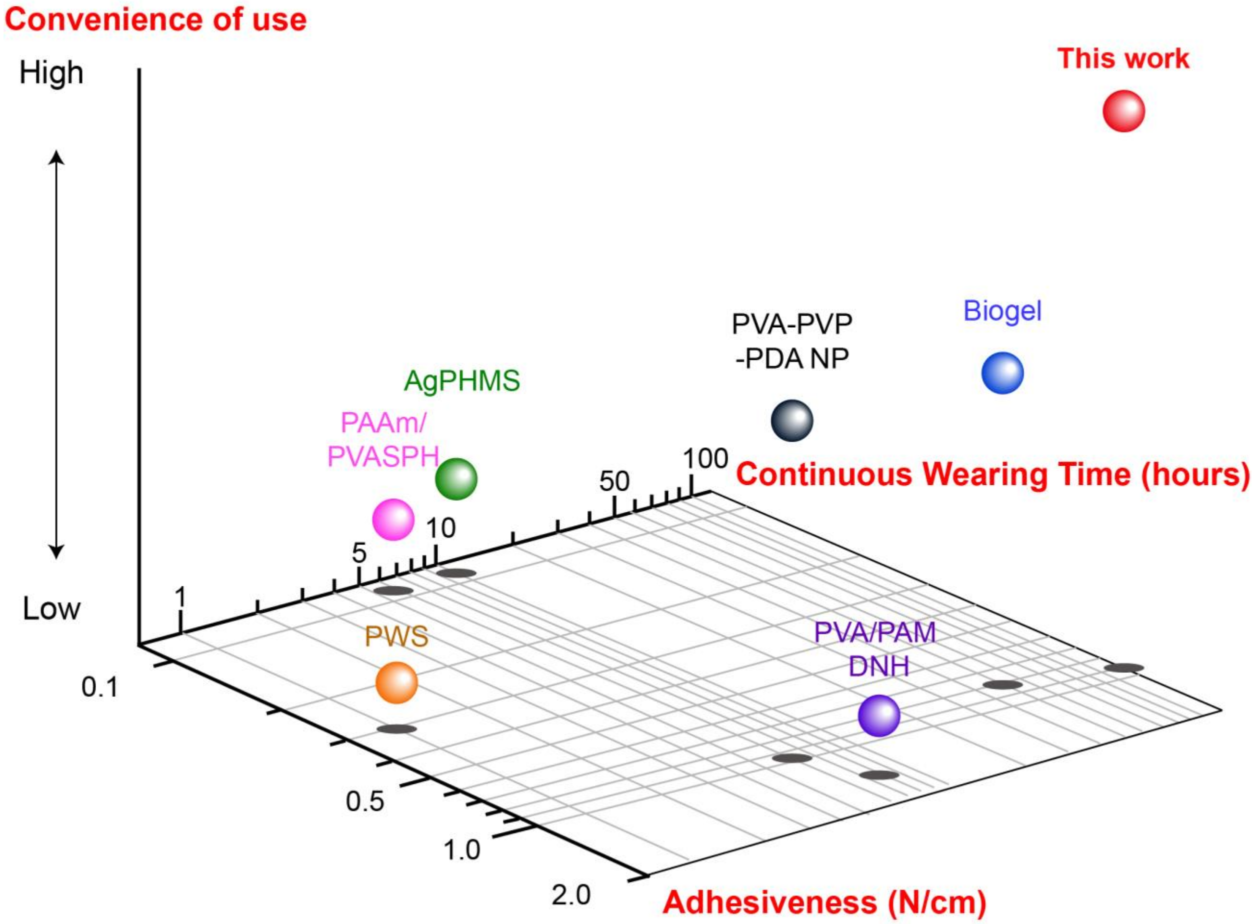

Figure S1. The properties of the AIRTrode hydrogel compared with those of previously reported hydrogels in terms of adhesiveness, continuous recording time, and convenience of use. This figure is related to Table S1.

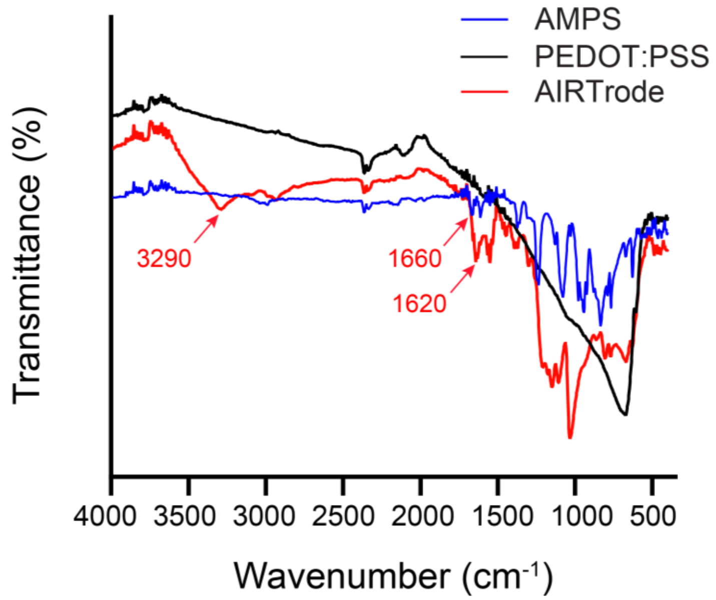

Figure S2. Fourier-Transform Infrared (FT-IR) spectra of AMPS (blue), PEDOT:PSS (black), and AIRTrode. FT-IR spectra were measured over a wavenumber range of 4000–400 cm^{-1}. This figure is related to Figure 1.

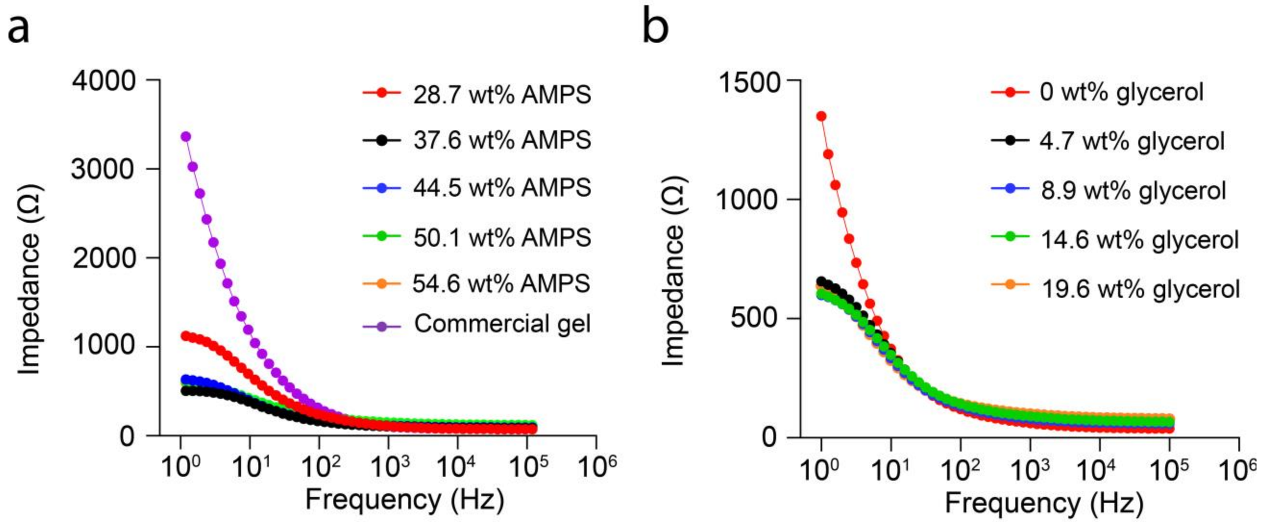

Figure S3. (**a**) Direct comparison of electrical impedance spectroscopy results between commercial EEG gel and the AIRTrodes with different AMPS loadings. (**b**) The influence of the presence of glycerol on the electrical impedance of AIRTrodes. Glycerol loading remained consistent at a 10 % weight ratio relative to PEDOT:PSS while adjusting AMPS loadings, and AMPS loading stayed consistent at a 100 % weight ratio relative to PEDOT:PSS while adjusting glycerol loadings. This figure is related to Figure 1.

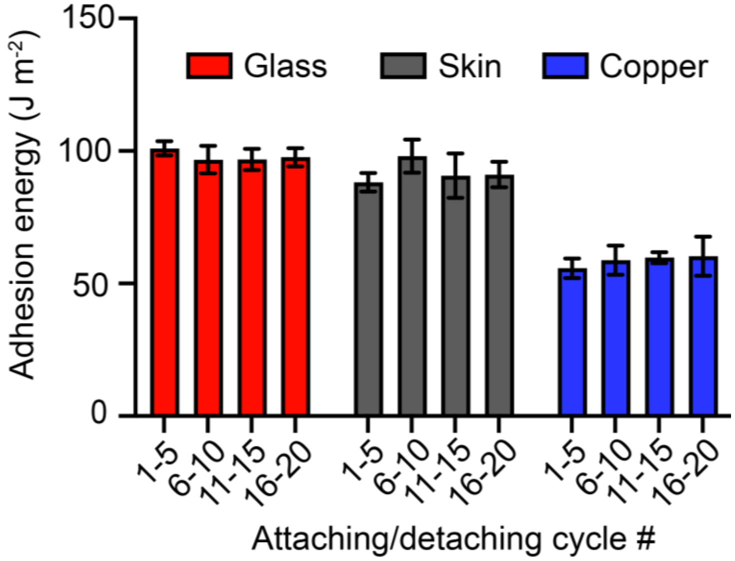

Figure S4. Stability of adhesion energy across 20 attaching/detaching cycles. This figure is related to Figure 2c.

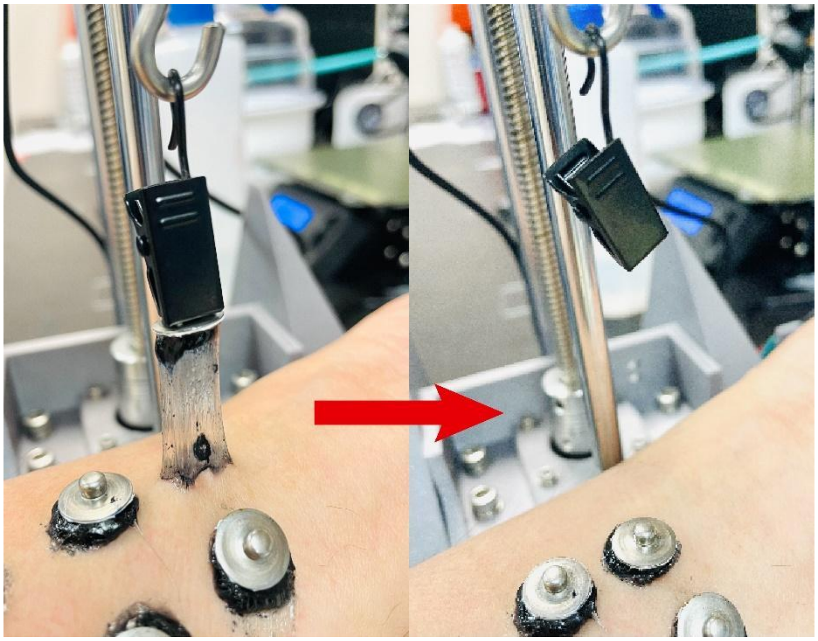

Figure S5. Illustration of the superior on-skin adhesion force and cohesion properties of AIRTrode. The AIRTrodes after pulling (left) could be reattached (right). This figure is related to Figure 2.

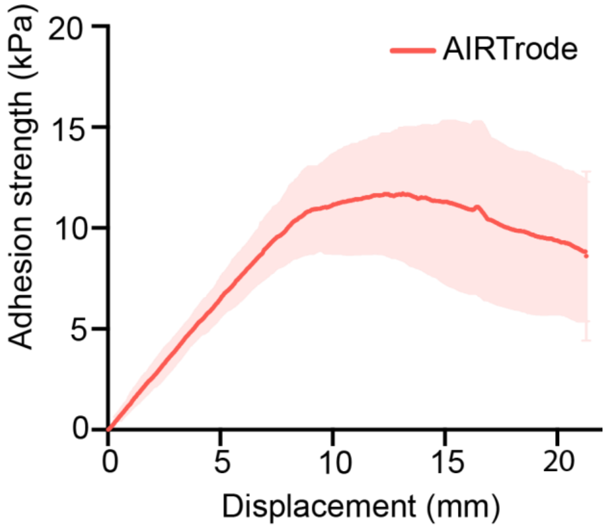

Figure S6. On-skin tensile adhesion force of AIRTrode. This figure is related to Figure 2.

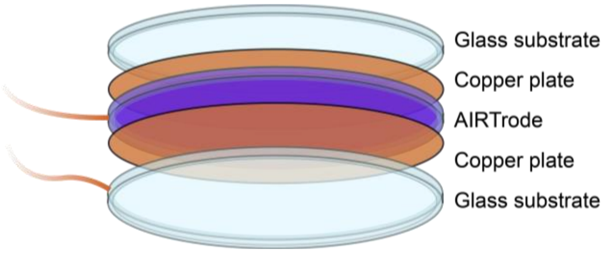

Figure S7. Schematic illustration of the electrical impedance measurement setup under prolonged open-air conditions. This figure is related to Figure 3.

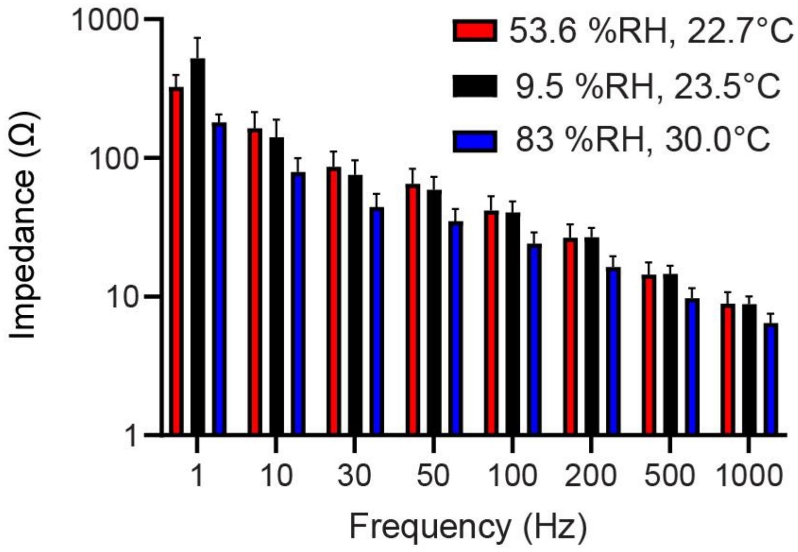

Figure S8. Impedance variation (1 Hz to 1000 Hz) of the AIRTrode electrodes under different relative humidity (RH) conditions. This figure is related to Figure 3.

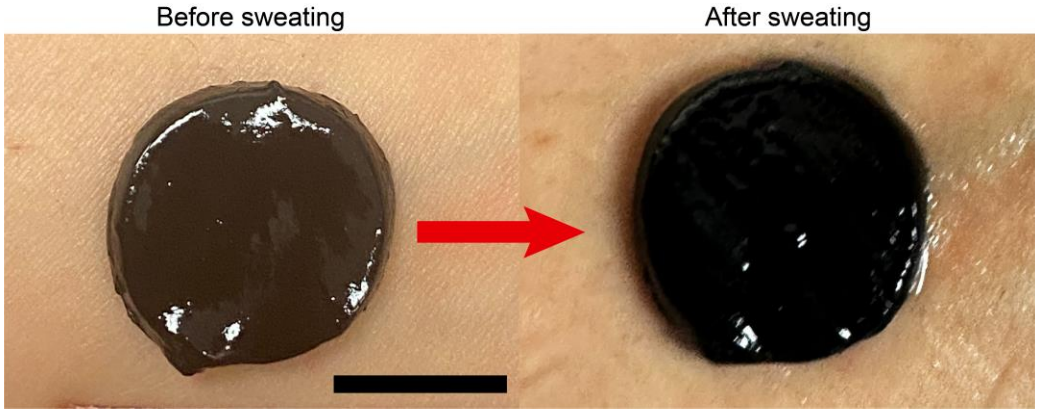

Figure S9. Stability on the sweating skin. AIRTrode electrodes were worn on the forearm when the subject went for an hour of outdoor walking at a high environmental temperature (32 ºC). AIRTrode electrodes can remain adhered to the skin before (left) and after (right) the experiment. The scale bar is 1 cm. This figure is related to Figure 3

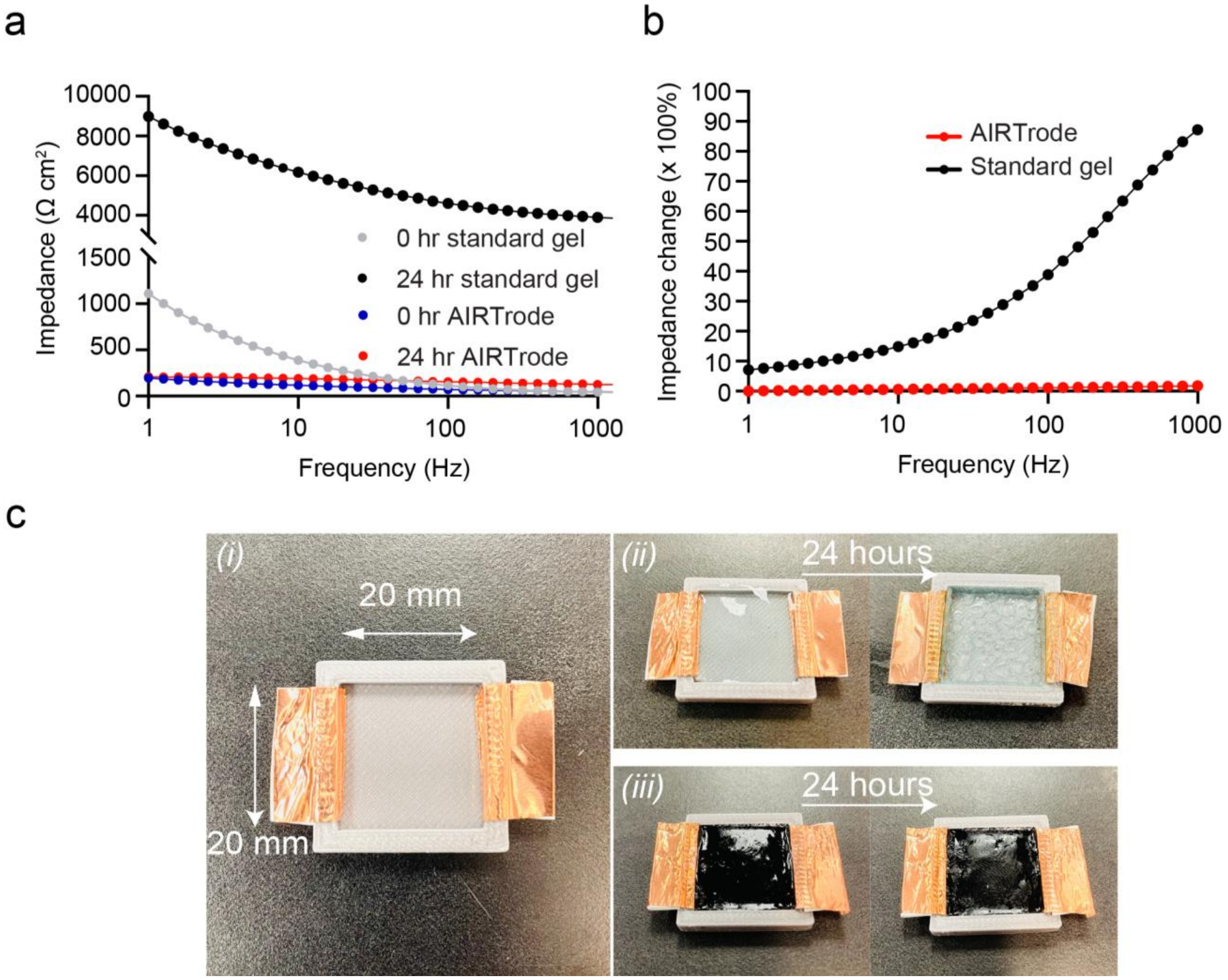

Figure S10. (**a**) Comparison of electrical impedance between commercial EEG gel and AIRTrode at 0 and 24 hours in the open-air condition. (**b**) Electrical impedance changes of commercial EEG gel and AIRTrode over 24 hours. (**c**) (i) Image of the 3D-printed cuboid tank for regulating the shape of the samples tested, (ii) the images of the tank with commercial EEG gel filled in at 0 hours and after 24 hours, and (iii) the same setting as (ii) for the tank with AIRTrode filled in. This figure is related to Figure 3.

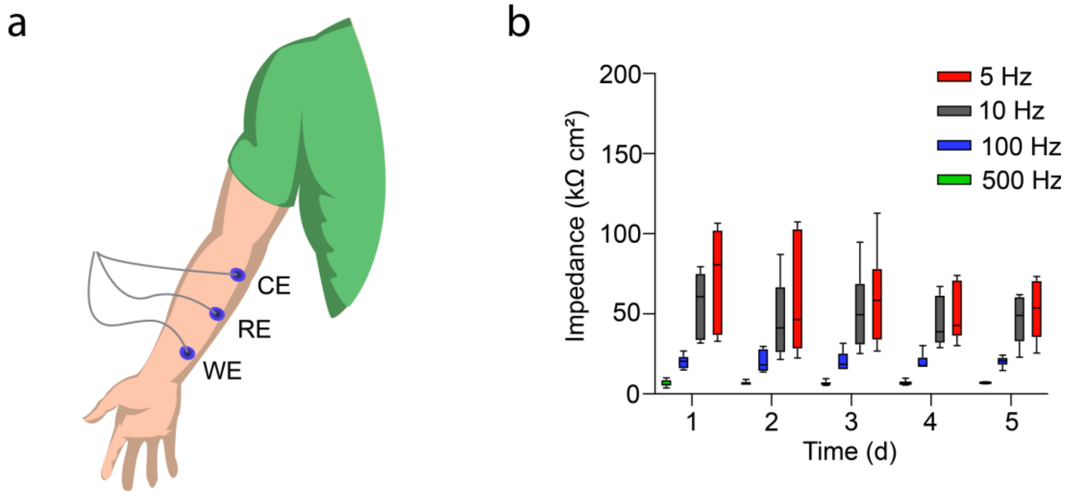

Figure S11. (**a**) Schematic illustration of standard three-electrode method of skin-electrode interfacial impedance measurement. (**b**) Averaged skin-electrode impedance from a cohort of participants (n = 6) across 5 days. This figure is related to Figure 3.

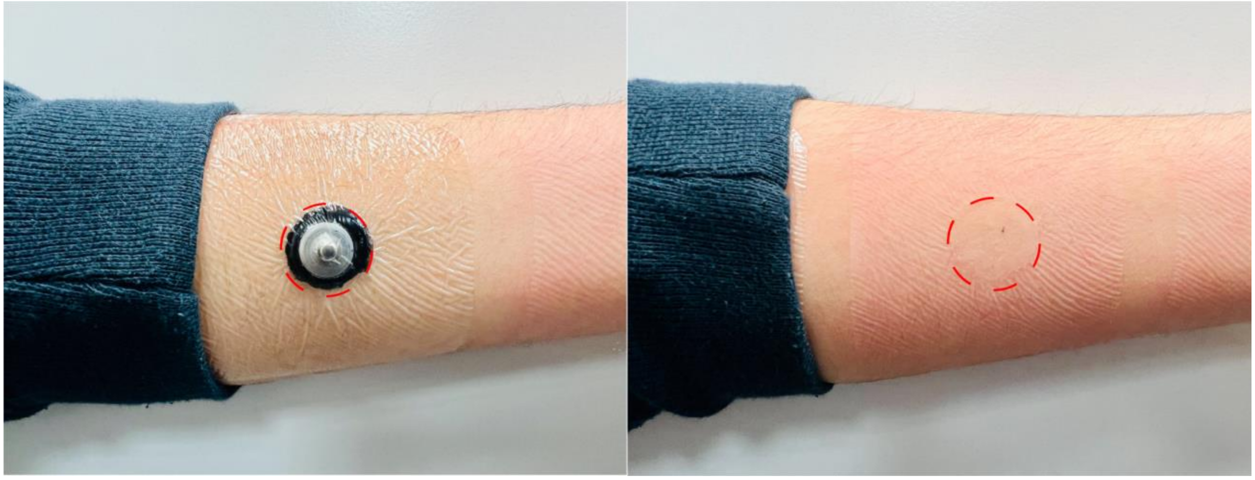

Figure S12. Zoom-in images depicting the state before (left) and after (right) electrode removal**.** This figure is related to Figure 3.

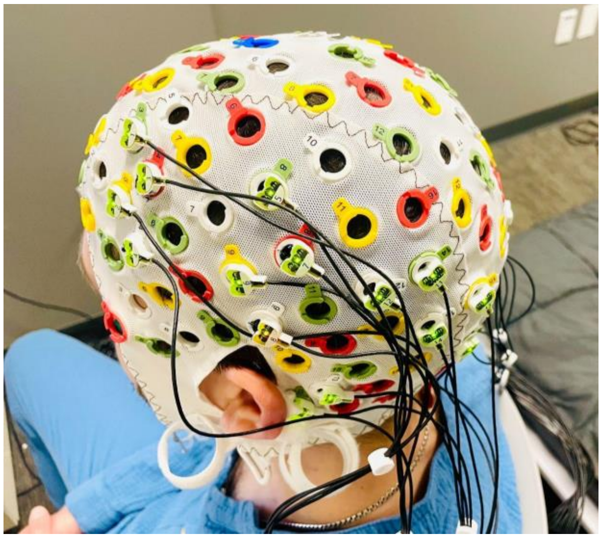

Figure S13. Image of a volunteer wearing an EEG cap with AIRTrode filled in during daytime (around 8 hours) while maintaining daily activities. This figure is related to Figure 3.

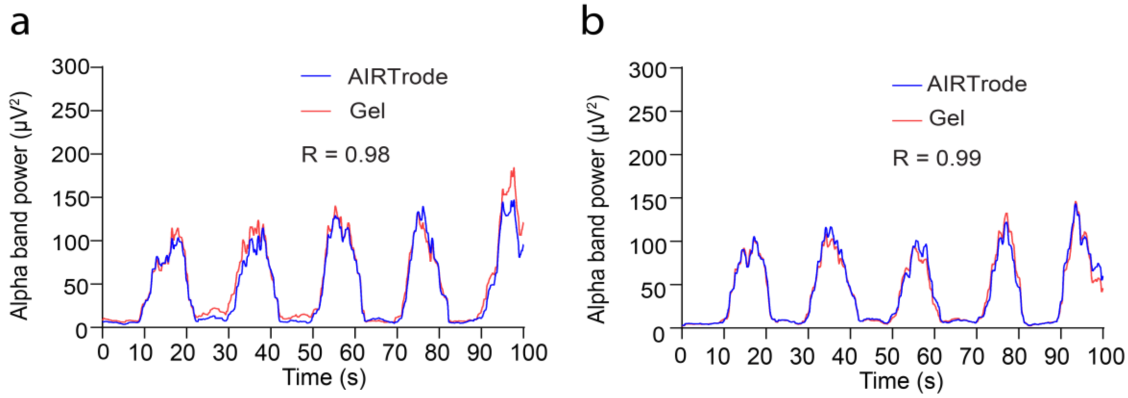

Figure S14. Time series alpha band ([8, 13] Hz) power from both electrodes (**a**) before overnight sleep and (**b**) after overnight sleep. This figure is related to Figure 4.

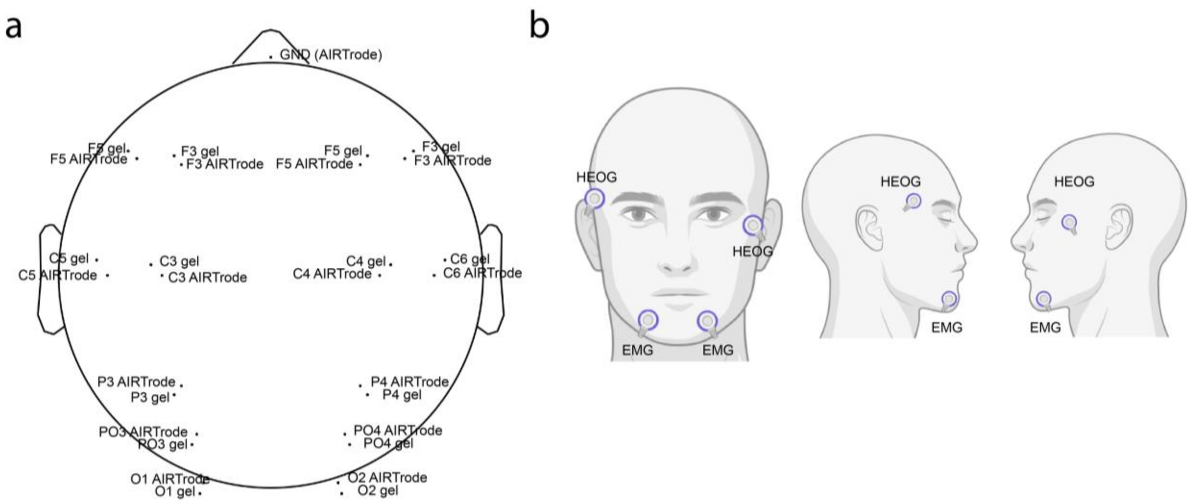

Figure S15. (**a**) Overnight sleep EEG montage setup. AIRTrode and commercial EEG gel electrodes for each recorded channel were placed at the proximity location to ensure the similarity of the source of the EEG signal. (**b**) Schematic illustration of the placements of the facial electrode pairs (HEOG and EMG). Figure S15b is created with BioRender.com. This figure is related to Figure 5.

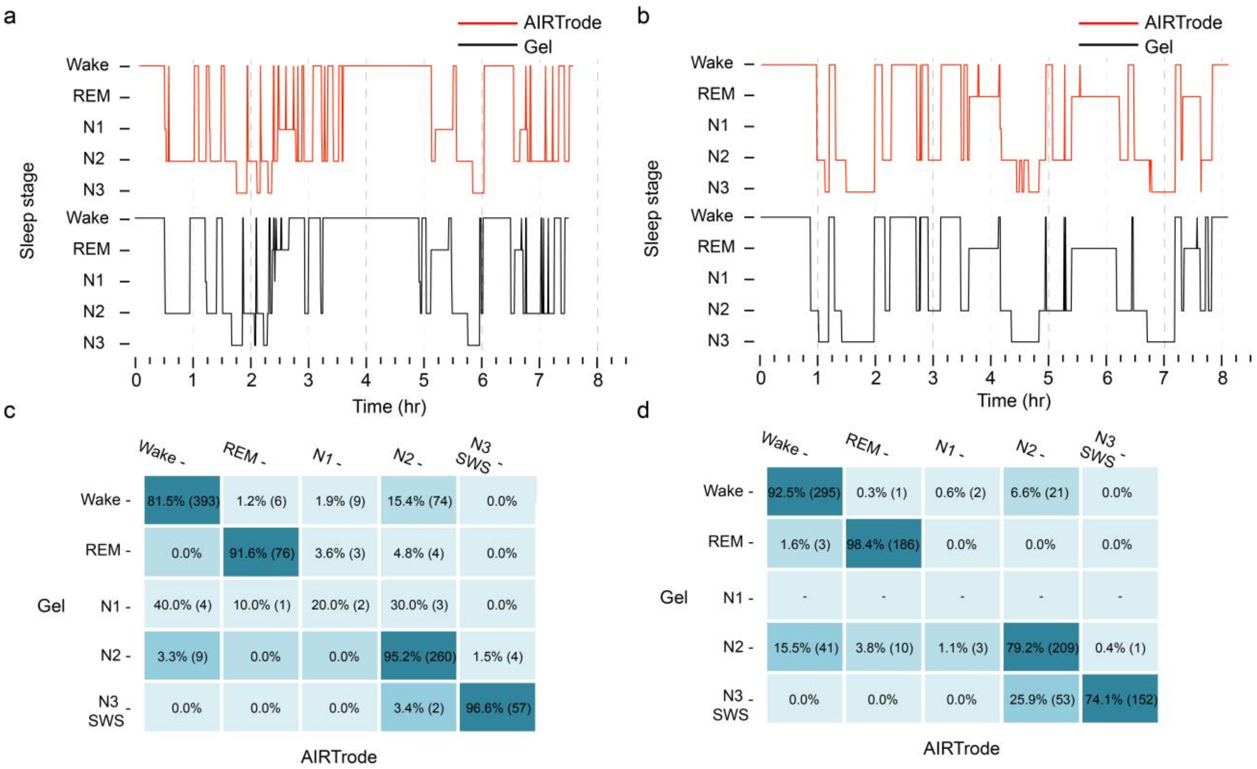

Figure S16. (**a, b**) Hypnograms and (**c, d**) the corresponding confusion matrices of the overnight sleep EEGs for two of the three participants. This figure is related to Figure 5.

Table S1. Comparison between AIRTrode and other hydrogel-based electrodes for long-term EEG applications.

Formation	Hydrogel	Young's modulus	Max Stretchability	Adhesion Strength	Skin Impedance[a] (10 Hz)	Continuous Wearing	Electrode location[b]	EEG applications	Require crosslink reagent/ treatment?	Require external fixation?
Preformed electrode	AIRTrode (preformed)	47.8 ± 11.6 kPa	>500%	0.92 ± 0.21 N cm^{-1}	31.70 kΩ cm^2	120 h	2	Sleep EEG	No	No
	AgPHMS[1]	58.6 kPa	N/A	N/A	5-15 kΩ	10 h	1	SSVEP, mVEP	.Yes	Yes
	PWS[2]	>30 MPa	43%	0.41 N cm^{-1}	82 kΩ cm^2	1 h (ECG)	1,2	Eyes open-closed paradigm	Yes	No
	NAGA hydrogel[3]	N/A	<375%	N/A	13.15 ± 3.72 kΩ cm^2	N/A	1,2	SSVEP, P300 visual	Yes	Yes
	Microstr.Ag LMP[4]	~ 1 MPa	N/A	~0.1 N cm^{-2}	50 kΩ cm^2	N/A	1	Eyes open-closed paradigm	Yes	Yes
	Soft ionic hydrogel[5]	N/A	N/A	N/A	17.40 kΩ (30 Hz)	3.5 h	1,2	SSVEP	Yes	Yes
	PVA/PAM DNH[6]	77.6 to 556 kPa	N/A	1.95 N cm^{-1}	20 ± 2.8 kΩ (after 8 h wearing)	8 h	1,2	Eyes open-closed paradigm/ P300/SSVEP	Yes	Yes
	PVA-PVP-PDA NP[7]	3.5–6.9 kPa	>800%	~ 1.0 N cm^{-1}	4.9 ± 0.1 kΩ	6 h (rat skin)	2 (forehead)	Attention evaluation	Yes	No
On-site formed electrode	AIRTrode (on-site formed)	17.1 ± 2.0 kPa	~300%	>0.92 ± 0.21 N cm^{-1}	17.50 ± 9.0 kΩ (after 8 h wearing)	>8 h (continuous recording)	1,2	Sleep EEG	No	No
	Biogel[8]	73.4 ± 2.2 kPa	~500%	0.80 ± 0.08 N cm^{-1}	6.95 ± 0.97 kΩ	48 h (1 test per day)	1,2	SSVEP	Yes	No
	PAAS-MXene hydrogel[9]	8 kPa	1088%	< 1.0 N cm^{-1}	<50 Ω (100 Hz)	N/A	1,2	EEG-based BMI	Yes	No
	PAAm/PVA SPH[10]	N/A	N/A	N/A	25.20 ± 6.92 kΩ*	6 h	1	SSVEP	Yes	Yes

[a]: skin preparation required

[b]:1 Hairy scalp, 2: Hairy-/non-hairy skin (ex: forearm)

Reference

1. Liu, J., Lin, S., Li, W., Zhao, Y., Liu, D., He, Z., Wang, D., Lei, M., Hong, B., and Wu, H. (2022). Ten-Hour Stable Noninvasive Brain-Computer Interface Realized by Semidry Hydrogel-Based Electrodes. Research *2022*, 9830457. 10.34133/2022/9830457.

2. Zhang, L., Kumar, K.S., He, H., Cai, C.J., He, X., Gao, H., Yue, S., Li, C., Seet, R.C.-S., Ren, H., et al. (2020). Fully organic compliant dry electrodes self-adhesive to skin for long-term motion-robust epidermal biopotential monitoring. Nat. Commun. *11*, 4683. 10.1038/s41467-020-18503-8.

3. Shen, G., Gao, K., Zhao, N., Yi, Z., Jiang, C., Yang, B., and Liu, J. (2021). A novel flexible hydrogel electrode with a strong moisturizing ability for long-term EEG recording. J. Neural Eng. *18*. 10.1088/1741-2552/ac41ab.

4. Stauffer, F., Thielen, M., Sauter, C., Chardonnens, S., Bachmann, S., Tybrandt, K., Peters, C., Hierold, C., and Vörös, J. (2018). Skin Conformal Polymer Electrodes for Clinical ECG and EEG Recordings. Adv. Healthc. Mater. *7*, e1700994. 10.1002/adhm.201700994.

5. Sheng, X., Qin, Z., Xu, H., Shu, X., Gu, G., and Zhu, X. (2021). Soft ionic-hydrogel electrodes for electroencephalography signal recording. Sci. China Tech. Sci. *64*, 273–282. 10.1007/s11431-020-1644-6.

6. Li, G., Liu, Y., Chen, Y., Li, M., Song, J., Li, K., Zhang, Y., Hu, L., Qi, X., Wan, X., et al. (2023). Polyvinyl alcohol/polyacrylamide double-network hydrogel-based semi-dry electrodes for robust electroencephalography recording at hairy scalp for noninvasive brain-computer interfaces. J. Neural Eng. *20*. 10.1088/1741-2552/acc098.

7. Han, Q., Zhang, C., Guo, T., Tian, Y., Song, W., Lei, J., Li, Q., Wang, A., Zhang, M., Bai, S., et al. (2023). Hydrogel nanoarchitectonics of a flexible and self-adhesive electrode for long-term wireless electroencephalogram recording and high-accuracy sustained attention evaluation. Adv. Mater. *35*, e2209606. 10.1002/adma.202209606.

8. Wang, C., Wang, H., Wang, B., Miyata, H., Wang, Y., Nayeem, M.O.G., Kim, J.J., Lee, S., Yokota, T., Onodera, H., et al. (2022). On-skin paintable biogel for long-term high-fidelity electroencephalogram recording. Sci Adv *8*, eabo1396. 10.1126/sciadv.abo1396.

9. Luo, J., Sun, C., Chang, B., Jing, Y., Li, K., Li, Y., Zhang, Q., Wang, H., and Hou, C. (2022). MXene-Enabled Self-Adaptive Hydrogel Interface for Active Electroencephalogram Interactions. ACS Nano *16*, 19373–19384. 10.1021/acsnano.2c08961.

10. Li, G., Wang, S., Li, M., and Duan, Y.Y. (2021). Towards real-life EEG applications: novel superporous hydrogel-based semi-dry EEG electrodes enabling automatically “charge-discharge” electrolyte. J. Neural Eng. *18*, 046016. 10.1088/1741-2552/abeeab.
